# Genome-wide association study of school grades identifies genetic overlap between language ability, psychopathology and creativity

**DOI:** 10.1038/s41598-022-26845-0

**Published:** 2023-01-09

**Authors:** Veera M. Rajagopal, Andrea Ganna, Jonathan R. I. Coleman, Andrea Allegrini, Georgios Voloudakis, Jakob Grove, Thomas D. Als, Henriette T. Horsdal, Liselotte Petersen, Vivek Appadurai, Andrew Schork, Alfonso Buil, Cynthia M. Bulik, Jonas Bybjerg-Grauholm, Marie Bækvad-Hansen, David M. Hougaard, Ole Mors, Merete Nordentoft, Thomas Werge, iPSYCH-Broad Consortium, Rich Belliveau, Caitlin E. Carey, Felecia Cerrato, Kimberly Chambert, Claire Churchhouse, Mark J. Daly, Ashley Dumont, Jacqueline Goldstein, Christine S. Hansen, Daniel P. Howrigan, Hailiang Huang, Julian Maller, Alicia R. Martin, Joanna Martin, Manuel Mattheisen, Jennifer Moran, Benjamin M. Neale, Jonatan Pallesen, Duncan S. Palmer, Carsten Bcker Pedersen, Marianne Giørtz Pedersen, Timothy Poterba, Stephan Ripke, F. Kyle Satterstrom, Wesley K. Thompson, Patrick Turley, Raymond K. Walters, Preben Bo Mortensen, Gerome Breen, Panos Roussos, Robert Plomin, Esben Agerbo, Anders D. Børglum, Ditte Demontis

**Affiliations:** 1 Department of Biomedicine, Aarhus University, Aarhus, Denmark; 2 The Lundbeck Foundation Initiative for Integrative Psychiatric Research (iPSYCH), Aarhus, Denmark; 3 Center for Genome Analysis and Personalized Medicine, Aarhus, Denmark; 4 Centre for Integrative Sequencing, iSEQ, Aarhus University, Aarhus, Denmark; 5 Institute for Molecular Medicine Finland, University of Helsinki, Helsinki, Finland; 6 Analytic and Translational Genetics Unit, Massachusetts General Hospital, Harvard Medical School, Boston, USA; 7 Broad Institute, Cambridge, USA; 8 Social, Genetic and Developmental Psychiatry Centre, Institute of Psychiatry, Psychology, and Neuroscience, King’s College London, London, UK; 9 National Institute of Health Research Maudsley Biomedical Research Centre, South London and Maudsley National Health Service Trust, London, UK; 10 Department of Psychiatry, Pamela Sklar Division of Psychiatric Genomics and Friedman Brain Institute, Icahn School of Medicine at Mount Sinai, New York, NY USA; 11 Department of Genetics and Genomic Sciences and Institute for Genomics and Multiscale Biology, Icahn School of Medicine at Mount Sinai, New York, NY USA; 12 James J. Peters VA Medical Center, Bronx, NY USA; 13 Bioinformatics Research Centre, Aarhus University, Aarhus, Denmark; 14 The National Centre for Register-Based Research (NCRR), Aarhus University, Aarhus, Denmark; 15 Institute of Biological Psychiatry, Mental Health Services of Copenhagen, Copenhagen, Denmark; 16 Neurogenomics Division, The Translational Genomics Research Institute (TGEN), Phoenix, AZ USA; 17 Department of Psychiatry, University of North Carolina at Chapel Hill, Chapel Hill, USA; 18 Department of Medical Epidemiology and Biostatistics, Karolinska Institutet, Stockholm, Sweden; 19 Department of Nutrition, University of North Carolina at Chapel Hill, Chapel Hill, USA; 20 Department for Congenital Disorders, Statens Serum Institut, Copenhagen, Denmark; 21 Psychosis Research Unit, Aarhus University Hospital-Psychiatry, Aarhus, Denmark; 22 Mental Health Center Copenhagen, Mental Health Services in The Capital Region of Denmark, Copenhagen, Denmark; 23 Department Clinical Medicine, Faculty of Health Science, University of Copenhagen, Copenhagen, Denmark; 24 Center for GeoGenetics, GLOBE Institute, University of Copenhagen, Copenhagen, Denmark; 25 Centre for Integrated Register-Based Research (CIRRAU), Aarhus University, Aarhus, Denmark; 26 Stanley Center for Psychiatric Research, Broad Institute of Harvard and MIT, Cambridge, MA USA; 27 Department of Psychology, Washington University in St. Louis, St. Louis, MO 63130 USA; 28 Genomics Plc, Oxford, UK; 29 Vertex Pharmaceuticals, Abingdon, UK; 30 MRC Centre for Neuropsychiatric Genetics and Genomics, Cardiff, University, Cardiff, UK; 31 Department of Psychiatry, Psychosomatics and Psychotherapy, University of Würzburg, Würzburg, Germany; 32 Department of Clinical Neuroscience, Karolinska Institutet, Stockholm, Sweden; 33 Department of Psychiatry and Psychotherapy, Charité-Universitätsmedizin, Berlin, Germany; 34 NORMENT-KG Jebsen Centre for Psychosis Research, University of Oslo, Oslo, Norway; 35 Division of Mental Health and Addiction, Oslo University Hospital, Oslo, Norway; 36 Behavioral and Health Genomics CenterCenter for Economic and Social Research, University of Southern, California, 635 Downey Way, Los Angeles, CA 90089 USA

**Keywords:** Behavioural genetics, Psychiatric disorders

## Abstract

Cognitive functions of individuals with psychiatric disorders differ from that of the general population. Such cognitive differences often manifest early in life as differential school performance and have a strong genetic basis. Here we measured genetic predictors of school performance in 30,982 individuals in English, Danish and mathematics via a genome-wide association study (GWAS) and studied their relationship with risk for six major psychiatric disorders. When decomposing the school performance into math and language-specific performances, we observed phenotypically and genetically a strong negative correlation between math performance and risk for most psychiatric disorders. But language performance correlated positively with risk for certain disorders, especially schizophrenia, which we replicate in an independent sample (n = 4547). We also found that the genetic variants relating to increased risk for schizophrenia and better language performance are overrepresented in individuals involved in creative professions (n = 2953) compared to the general population (n = 164,622). The findings together suggest that language ability, creativity and psychopathology might stem from overlapping genetic roots.

## Introduction

Psychiatric disorders are common and have a complex aetiology with contributions from both genetic and environmental factors^1^. They are typically characterized by an early age of onset^2^. While some disorders emerge during childhood, for example, autism spectrum disorder (ASD) and attention deficit hyperactivity disorder (ADHD), some emerge during adolescence or early adulthood, for example, schizophrenia (SCZ), bipolar disorder (BD) and anorexia nervosa (AN). Even long before the symptoms manifest, individuals often show signs of psychopathology^3^. Several epidemiological studies have found atypical school performance as a risk factor for psychiatric disorders. Poor school performance has been shown as a risk factor for SCZ^3^ whereas excellent school performance for BD^4^. Atypical school performance has been reported also in unaffected children and siblings of psychiatric patients suggesting that genetic factors could be involved^5,6^.

Large-scale GWASs have been conducted for many major psychiatric disorders to date^7–12^, demonstrating a complex genetic architecture and strong genetic correlations with a broad range of phenotypes, including cognitive phenotypes such as educational attainment^13^ and intelligence^14^. We have previously reported a strong negative genetic correlation between ADHD^7^ and educational attainment and a moderate positive genetic correlation between ASD^8^ and educational attainment in the largest GWASs of ADHD^7^ and ASD^8^ respectively. Likewise, a GWAS has reported a strong positive genetic correlation between AN and educational attainment^12^.

For most of the psychiatric disorders, the genetic correlations with educational attainment align with the corresponding phenotypic associations with school performance reported in epidemiological studies^15–17^. For some disorders such as SCZ, however, the genetic correlations contradict phenotypic associations^18^. Although clinical and epidemiological studies have documented that individuals with—or at risk for—SCZ perform poorly in school^19^ and score low in neurocognitive assessments^20^, the genetic correlation of SCZ with educational attainment is not negative, but rather positive, albeit weak^18^. Similarly, although BD has been shown to be associated with cognitive deficits^21^, it shows a positive genetic correlation with educational attainment^10^. Such findings indicate that heterogeneity exists in the genetic overlap between psychiatric disorders and cognitive phenotypes. The current large GWASs of educational attainment^13^ and intelligence^14^ do not inform about what causes such heterogeneities. This could be due to that educational attainment and intelligence are composite measures and capture multiple cognitive domains each of which correlates differently with psychiatric disorders (some correlating positively and some negatively). Also, it has been suggested that the cognitive component in educational attainment that correlates positively with disorders such as SCZ and BD might represent creativity^22^, as creativity has been shown to associate positively with these disorders^21,22^. However, it is not clear which cognitive domain specifically corresponds to creativity. Given these backgrounds, performing GWASs of specific cognitive domains and studying their genetic associations with psychiatric disorders and creativity might offer significant insights into the complex relationship between educational attainment, psychiatric risk and creativity.

Compared to educational attainment, school grades offer fine-grained information. For example, grades in language and math subjects might serve as proxies for verbal and numerical cognition respectively. However, no large-scale GWASs of subject-specific school grades have been conducted so far. The existing GWASs of school grades were based on small sample sizes insufficient to study genetic overlap with the psychiatric disorders^23,24^.

Here we present the largest GWAS of school grades to date disentangling the polygenic architecture of various domains of school performance and their complex phenotypic and genetic relationships with six major psychiatric disorders (ADHD, ASD, SCZ, BD, major depressive disorder (MDD) and AN). The overall study design is shown in Supplementary Fig. 1. Our discovery sample comes from iPSYCH^25^ and the Anorexia Nervosa Genetics Initiative (ANGI)^26^, large population-based Danish cohorts of individuals with and without psychiatric disorders for whom information on school grades was available through the Danish education register^27^. Using principal component analysis (PCA), we decomposed the school grades in Danish, English and mathematics into orthogonal principal components (hereafter, E-factors) thereby capturing distinct cognitive domains. The GWASs of the E-factors (E1, E2, E3 and E4) identified multiple genome-wide significant loci. Among the E-factors, E1 correlated the most with educational attainment^28^ and intelligence^14^. E2 captured differences between language and math performances and showed positive phenotypic and genetic correlations with almost all the psychiatric disorders, thereby shedding light on the differential relationship of psychiatric disorders with math and language cognitive domains. In addition, E2 showed a positive genetic association with creativity, suggesting a shared genetic basis between language ability and creativity.

## Results

### Sample characteristics

Totally 30,982 individuals from iPSYCH and ANGI were studied, of which 18,495 (60%) had a diagnosis for at least one of the six psychiatric disorders, and 12,487 (40%) did not have any. The sample characteristics are shown in Table 1. The age of the study individuals (as of Dec 2016) ranged between 15 to 38 years (mean = 24.2; SD = 4.2). There were 14,606 males and 16,376 females. The school grades were from the exit exam (or ninth-level exam or FP9) given at the end of compulsory schooling in Denmark. Age at the time of examination (hereafter, exam age) ranged between 14.5 and 17.5 years (mean = 15.7; SD = 0.42). Grades in three subjects namely Danish, English and mathematics were analyzed. Individuals with psychiatric diagnoses (as of Dec 2016) were considered as cases irrespective of whether the diagnoses were given before or after the exit exam.

**Table 1:** Sample characteristics.

Variable	SCZ	BD	MDD	ADHD	ASD	AN	Controls
Sample size	1356	839	9719	5238	3859	1680	12,487
Females	668 (49.3%)	561 (66.9%)	6836 (70.3%)	1748 (33.4%)	1017 (26.4%)	1568 (93.3%)	6327 (50.7%)
Age on Dec 2016	26.1 (2.9)	26.8 (2.8)	26.2 (3.3)	22.9 (4.1)	22.1 (3.9)	24.8 (3.7)	23.4 (4.2)
Exam age^a^	15.7 (0.4)	15.6 (0.4)	15.6 (0.4)	15.8 (0.4)	15.8 (0.5)	15.6 (0.4)	15.6 (0.4)
Age at first diagnosis^b^	21.15 (2.9)	21.86 (3.2)	18.9 (3.3)	14.8 (5.6)	12.8 (5.0)	16.3 (3.3)	–
Diagnosed before exam^c^	38 (2.8%)	17 (2.1%)	1655 (17.1%)	3025 (57.8%)	2880 (74.6%)	819 (48.9%)	–
Mean school grades^d^	6.0 (2.2)	6.8 (2.3)	6.4 (2.3)	5.3 (2.2)	6.7 (2.3)	8.0 (2.3)	6.9 (2.4)

The values represent either frequency (proportion) if categorical variable or mean (SD) if continuous variable.

^a^Exam age is the age of the individuals when they sat for the exit exam.

^b^The age at first diagnosis was calculated based on the first time the diagnosis was recorded in the register.

^c^The date of first diagnosis and the date of exit exam were compared to identify if the individual has received the diagnosis before they sat for the exam.

^d^The mean value was calculated across all the grades: Danish written, oral and grammar and English oral and mathematics written and oral (if sat for the exam before on or before 2006) or problem solving (if sat for the exam after 2006); The lowest and highest mean values observed were − 1.7 and 12.

### Decomposition of school grades into cognitive domains

**Figure 1:**
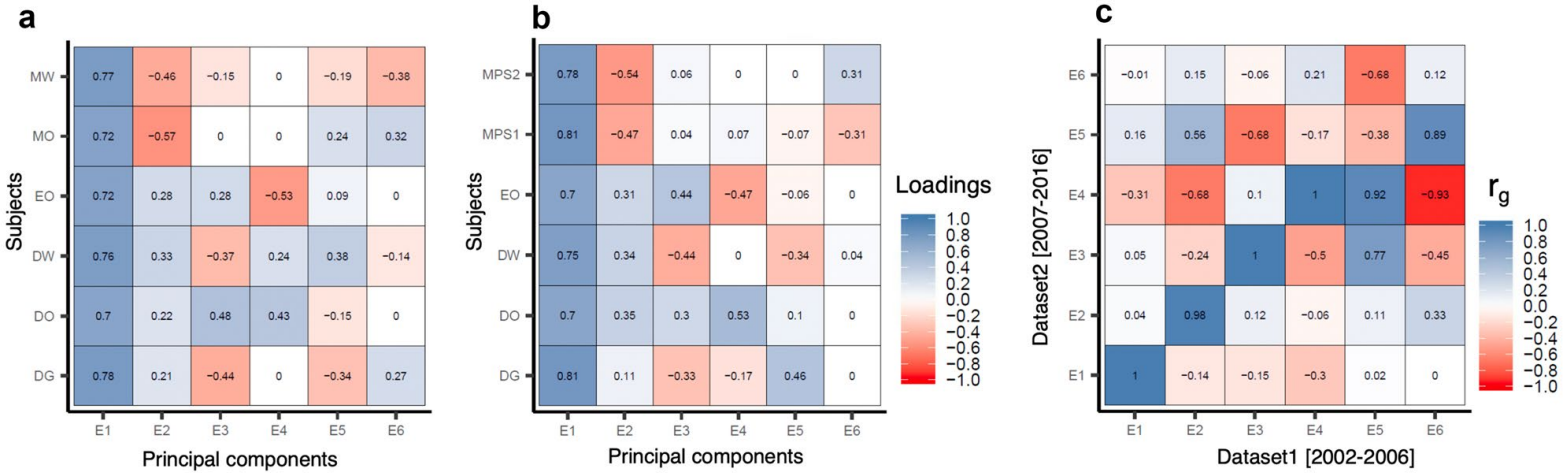
PC loadings and genetic correlations of E-factors. (**a**) Pearson correlations between the first 6 PCs and subject-specific grades in dataset 1 (i.e., based on exams conducted between 2002 and 2006; N=11,284); (**b**) Pearson correlations between the first 6 PCs and subject-specific grades in dataset 2 (i.e., based on exams conducted between 2007 and 2016; N=19,698); (**c**) Pairwise genetic correlations (r_g_) between dataset 1 PCs and dataset 2 PCs estimated using GCTA bivariate GREML analysis; *DG* Danish grammar, *DO* Danish oral, *DW* Danish written, *EO* English oral, *MO* Math oral, *MW* Math written, *MPS1* Math problem solving 1 (with help e.g., by using a calculator), *MPS2* Math problem solving 2 (without help).

For each individual we had information on the following grades: Danish written, oral and grammar, English oral, mathematics written and oral (if sat for the exam before 2007), mathematics problem solving with help and problem solving without help (if sat for the exam after 2006; “Methods”). All the grades individually showed substantial heritability and strongly correlated with each other, both phenotypically and genetically (Supplementary Fig. 2). Individual GWASs of these grades may yield largely similar results and may inform little about the genetic overlap of distinct performance domains with psychiatric disorders. Hence, we decomposed the grades into latent factors (E-factors) that might represent distinct cognitive domains, using a principal component analysis (PCA). Since the mathematics exams were restructured in 2007, we performed PCA separately for grades given during 2002–2006 and 2007–2016 (“Methods”). We identified four informative E-factors that were reproducible between the two PCAs in terms of subject loadings and showed near-perfect genetic correlations (rg ~ 1) between the two datasets (Fig. 1a–c and Supplementary Table 1). After combining the two datasets, the four E-factors together explained 89.5% of the variance in the school grades (E1 = 56%; E2 = 13.5%; E3 = 10.5%; E4 = 8.5%).

We discuss in detail how to interpret the four E-factors based on their subject-specific loadings in the Supplementary Note. Briefly, E1 captured overall school performance (analogous to general cognitive ability factor (g) derived from a battery of cognitive tests^29^), E2 captured language performance relative to math, E3 captured oral performance relative to written, and E4 captured Danish performance relative to English (Table 2). We also repeated the PCA only in the control individuals (N = 12,487) and found similar subject loadings to that of the main PCA (Supplementary Fig. 3). Hence, the subject loading pattern in the main PCA was not biased due to the inclusion of individuals with psychiatric disorders.

**Table 2:** Description of E-factors.

E-factors	Variance^a^ (%)	Description
E1	56	Captures overall school performance as the loadings were similar across all subjects; higher the E1 score, better the individual in all the grades
E2	13.5	Captures the difference between language and math performance; higher the E2 score, better the individual in language relative to math
E3	10.5	Captures the difference between oral and written grades in Danish and English; higher the E3 score, better the individual in oral exams relative to written exams
E4	8.5	Captures the difference between Danish and English performances; higher the E4 score, better the individual in Danish relative to English

^a^The variance estimate is the proportion of the variance explained individually by the E-factors in the school grades.

The factors E2, E3 and E4 all capture only the relative performance hence should be interpreted carefully. For example, E2 informs only if an individual is better in language relative to math. E2 score can be high even when the individual is poor overall in both language and math (in comparison to others), but worse in math compared to language.

### GWAS of E-factors

We performed GWASs for the four E-factors using a genetically homogenous sample of unrelated Europeans that comprised both individuals with and without psychiatric disorders (“Methods”). Psychiatric diagnoses as well as sex and exam age were included in the covariates as they were all significantly associated with the E-factors (“Methods”; Supplementary Table 2 and Supplementary Note).

We identified seven genome-wide significant loci (P < 5 × 10^–8^), of which four were associated with E1, two with E2, one with E3 and none with E4 (Supplementary Table 3; Supplementary Fig. 4; Supplementary Dataset 1). Among these, only three remained strictly significant (P < 1.2 × 10^–8^) after adjusting for the four GWASs conducted. In a phenome-wide association study (see “Methods”) of the index variants in the seven loci, six were significantly associated with multiple cognitive phenotypes (Supplementary Table 4; Supplementary Dataset 2). Biological annotations of the seven loci are discussed in the Supplementary Note. The SNP-based heritability estimates were as follows: E1 = 0.29 (SE = 0.01; P < 1.0 × 10^–300^), E2 = 0.18 (SE = 0.01; P < 1.0 × 10^–300^), E3 = 0.13 (SE = 0.01; P < 1.0 × 10^–300^) and E4 = 0.08 (SE = 0.01; P = 1.0 × 10^–13^). We observed moderate levels of inflation in the GC lambda values, which were likely due to polygenicity rather than biases such as population stratification and cryptic relatedness as suggested by LD score regression analysis^30^ (Supplementary Table 5).

### Association of E-factors with educational attainment and intelligence

**Figure 2:**
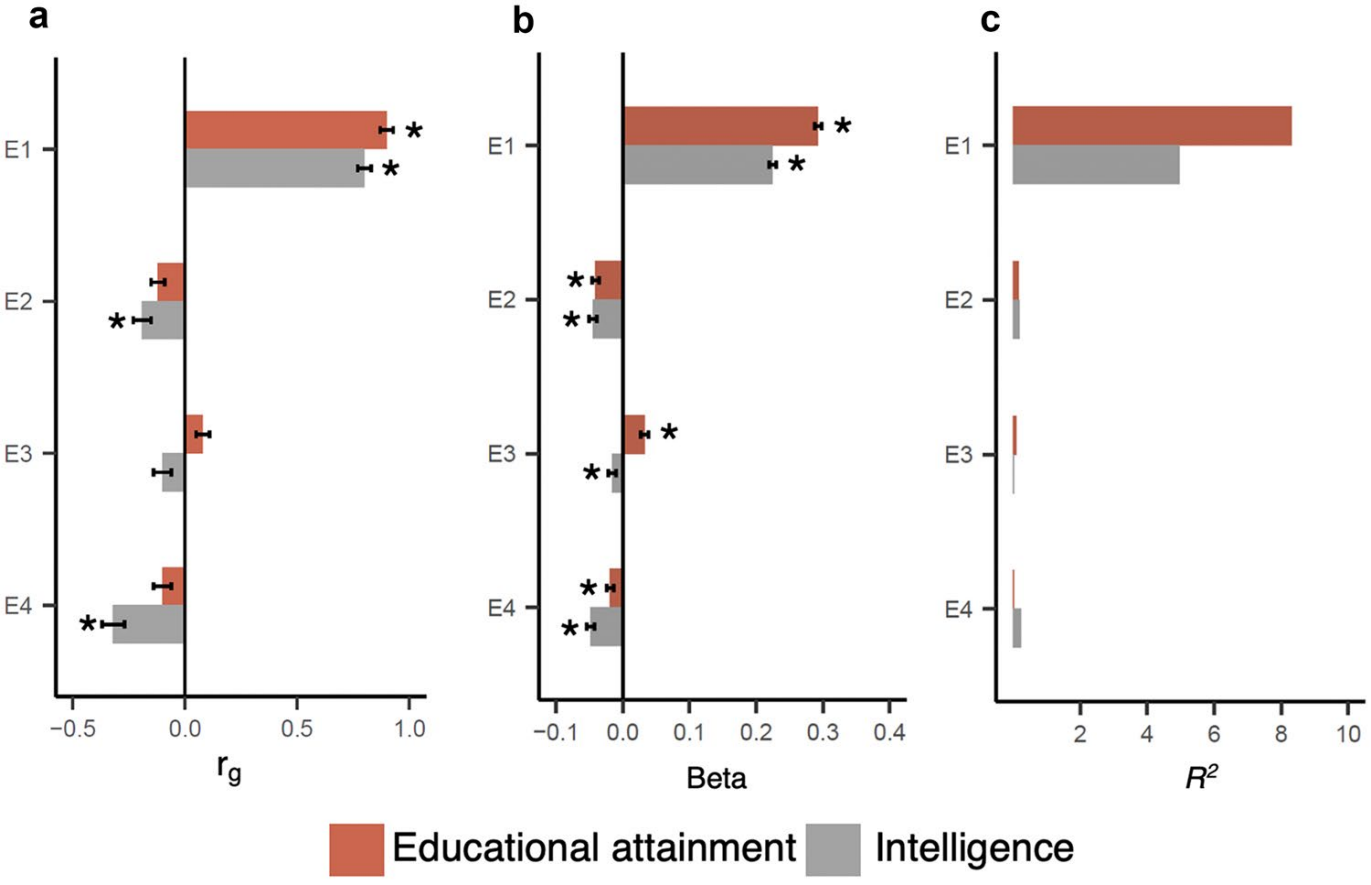
Genetic associations of E-factors with educational attainment and intelligence. (**a**) Genetic correlations (r_g_) of the E-factors with educational attainment and intelligence estimated using bivariate LD score regression; (**b**,**c**) Associations of polygenic scores for educational attainment and intelligence with the E-factors. Standardised effect sizes (beta coefficient) are shown in (**b**) and variances explained (R^2^) are shown in (**c**). Star symbols indicate statistical significance after multiple testing corrections (P < 0.006).

Among the four E-factors, E1 captured overall school performance and showed the strongest genetic correlations with educational attainment^28^ (r_g_ = 0.90; SE = 0.03; P = 4.8 × 10^–198^) and intelligence^14^ (r_g_ = 0.80; SE = 0.03; P = 3.3 × 10^–128^) (Fig. 2a; Supplementary Table 6). We also studied the associations of polygenic scores for educational attainment and intelligence (“Methods”) with the E-factors. The polygenic scores showed strong associations with E1 and explained 8.3% (educational attainment) and 4.9% (intelligence) of the variance in E1 (Fig. 2b,c; Supplementary Table 7). The genetic associations of educational attainment and intelligence with E2, E3, and E4 were only modest (Fig. 2; Supplementary Tables 6, 7; Supplementary Note).

### Phenotypic and genetic associations of E-factors with psychiatric disorders

Next, we evaluated the phenotypic and genetic associations of the E-factors with six psychiatric disorders (ADHD^7^, ASD^8^, SCZ^31^, BD^32^, MDD^11^ and AN^12^). Phenotypic associations were evaluated by comparing the E-factor scores of each psychiatric disorder group against the controls. Genetic associations were evaluated using two approaches. First, we studied the genetic correlations of the E-factors with the six psychiatric disorders using LD score regression. Second, we constructed polygenic scores for the six psychiatric disorders in the iPSYCH cohort using variant effect sizes from past psychiatric GWASs and studied their associations with the E-factors only in the controls.

**Figure 3:**
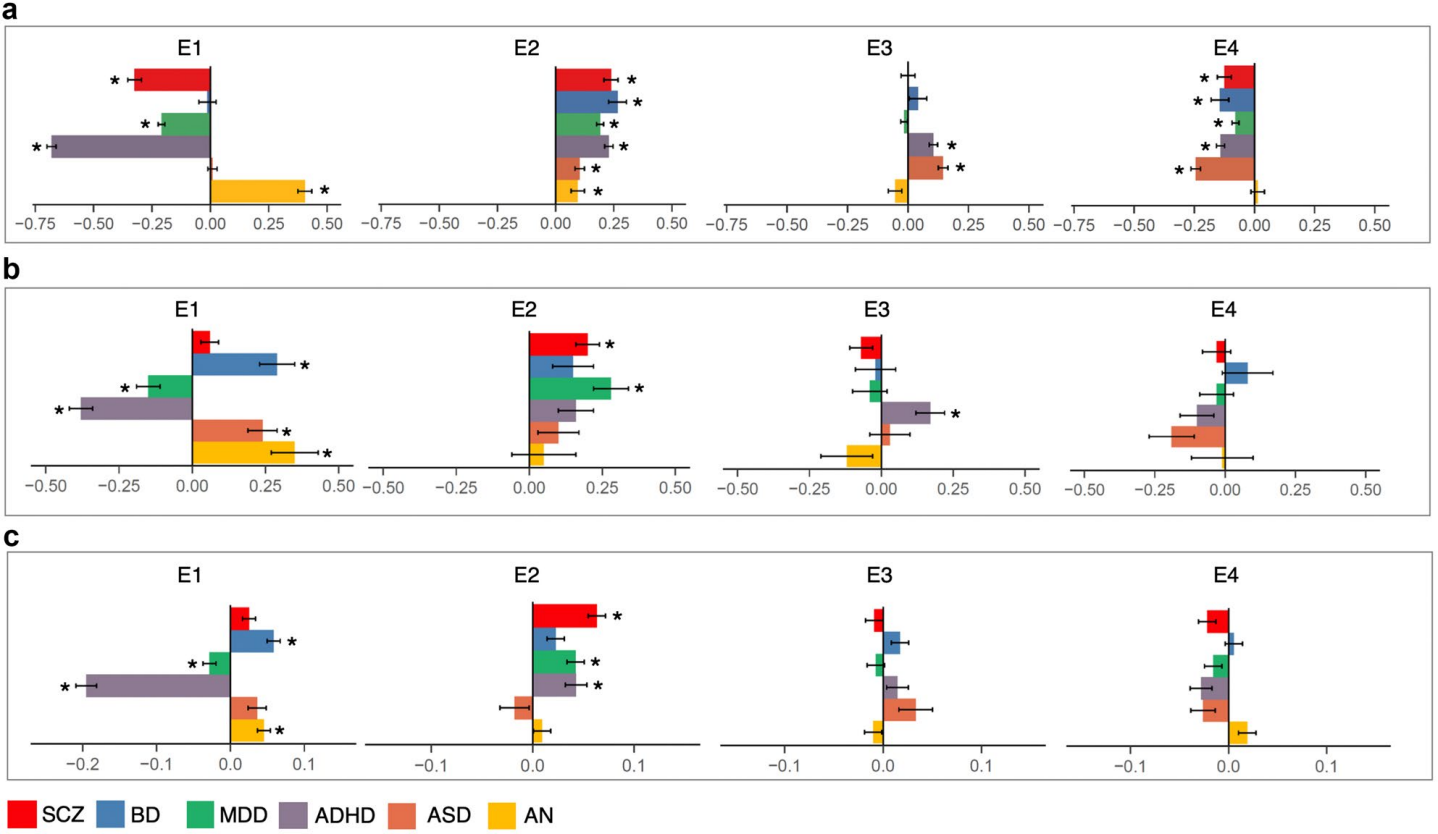
Phenotypic and genetic associations of E-factors with psychiatric disorders. (**a**) Phenotypic associations of the E-factors with the six psychiatric disorders tested using logistic regression. Standardised effect sizes (beta coefficient) along with standard errors are shown; (**b**) Genetic correlations (r_g_) of the E-factors with the six psychiatric disorders estimated using bivariate LD score regression; (**c**) Associations of polygenic scores for the six psychiatric disorders with the E-factors analyzed only in the controls (N = 12,487). Beta coefficients and standard errors are shown. Star symbols indicate statistical significance after multiple testing corrections (P < 0.002). *SCZ* schizophrenia, *BD* bipolar disorder, *MDD* major depressive disorder, *ADHD* attention deficit hyperactivity disorder, *ASD* autism spectrum disorder, *AN* anorexia nervosa.

E1 (overall school performance) showed strong phenotypic associations with four out of the six psychiatric disorders analyzed (Fig. 3a; Supplementary Table 8). The average E1 scores of ADHD, MDD and SCZ were significantly lower than controls and that of AN was significantly higher than controls. The average E1 scores of ASD and BD did not differ significantly from the controls. E1 showed significant negative genetic correlations with ADHD and MDD, but not with SCZ (Fig. 3b; Supplementary Table 9). Despite having a strong negative phenotypic association with SCZ, E1 showed a weak positive genetic correlation with SCZ, which was not statistically significant. Finally, E1 showed significant positive genetic correlations with ASD, BD and AN. Associations of polygenic scores for the six psychiatric disorders with the E-factors only in the controls recapitulated the genetic correlation findings (Fig. 3c; Supplementary Table 10).

E2 (language performance relative to math) showed significant phenotypic associations with all six psychiatric disorders (Fig. 3a; Supplementary Table 8). The average E2 scores of all six disorder groups were substantially higher than controls (meaning cases performed better in language relative to math compared to controls). Similar to the phenotypic associations, E2 showed positive genetic correlations with all six disorders (Fig. 3b; Supplementary Table 9), though statistical significance was achieved only for SCZ (r_g_ = 0.20; SE = 0.04; P = 2.1 × 10^–5^) and MDD (r_g_ = 0.28; SE = 0.06; P = 1.1 × 10^–5^). Associations of psychiatric polygenic scores only in the controls recapitulated the genetic correlation findings (Fig. 3c; Supplementary Table 10). The results overall suggested that a better performance in language relative to math was seen in all six psychiatric disorders and this differential performance seems to have a strong genetic basis, particularly in SCZ and MDD.

Although E3 (oral performance relative to written) and E4 (Danish performance relative to English) showed significant phenotypic associations with many of the psychiatric disorders, these associations were not supported by the genetic correlation and polygenic score analysis, which could be due to a lack of power as they explained relatively smaller amounts of variances in the school grades (Fig. 3; Supplementary Tables 8, 9, 10; Supplementary Note).

### Association of language and math performances with psychiatric disorders

While the interpretation of E1 findings is straightforward, the interpretation of E2 findings is not. Observing higher E2 scores in cases compared to controls does not always imply that language grades in cases are higher than controls and math grades are lower than controls. Even when both the language and math grades are lower in cases than in controls, the E2 scores in cases can be still higher than controls, if the language grades of cases are relatively higher than their math grades. Thus, to gain clarity on the relationship of E2 with the six psychiatric disorders, we compared the actual math and language grades between cases and controls. We calculated the mean across all math grades and the mean across all Danish and English grades for each individual and used them as measures of math and language performances respectively. Using a multiple logistic regression analysis (adjusted for exam age and sex), both math and language grades were included in the same regression model, thereby testing if the language performance differed between cases and controls after controlling for the differences in the math performance and vice versa.

**Figure 4:**
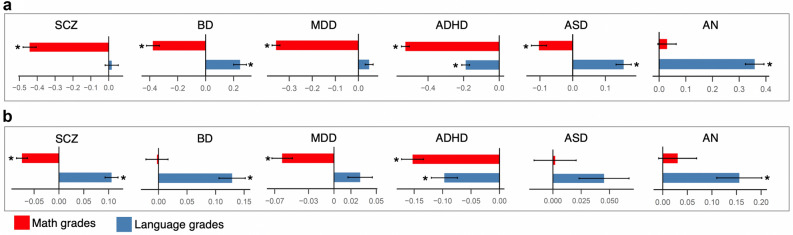
Phenotypic and genetic associations of math and language grades with psychiatric disorders. (**a**) Phenotypic associations of math and language grades with the six disorders tested using multiple logistic regression; standardized effect sizes (beta) and standard errors are shown; (**b**) Associations of polygenic scores for the six psychiatric disorders with math and language grades analyzed only in the controls (N = 12,487); standardized effect sizes (beta) and standard errors are shown. Star symbols indicate statistical significance after multiple testing corrections (P < 0.004). *SCZ* schizophrenia, *BD* bipolar disorder, *MDD* major depressive disorder, *ADHD* attention deficit hyperactivity disorder, *ASD* autism spectrum disorder, *AN* anorexia nervosa.

For all six disorders, we observed substantial differences between math and language performances in cases compared to controls (Fig. 4a; Supplementary Table 11). At the phenotypic level, math grades were significantly lower in cases compared to controls for all disorder groups except AN (Fig. 4a; Supplementary Table 11). Notably, among all the disorder groups, the SCZ cases scored on average lowest in math compared to controls (Beta = − 0.44; SE = 0.03; P = 3 × 10^–33^). In contrast to math grades, the language grades were significantly higher in cases compared to controls in BD (Beta = 0.24; SE = 0.04; P = 8.5 × 10^–8^), ASD (Beta = 0.15; SE = 0.02; P = 2 × 10^–11^) and AN (Beta = 0.35; SE = 0.03; P = 7.4 × 10^–25^). For ADHD, the language grades (Beta = − 0.18; SE = 0.02; P = 1 × 10^–17^) were lower in cases compared to controls, though the difference was only less than half of the math difference (Beta = − 0.52; SE = 0.02; P = 2.4 × 10^–123^). For SCZ (Beta = 0.01; SE = 0.03; P = 0.64) and MDD (Beta = 0.04; SE = 0.01; P = 0.006; adjusted P = 0.07), no statistically significant differences were seen in the language grades between cases and controls.

Next, we asked if the impact of psychiatric disorders on language-math performance differences could extend beyond cases to the general population. That is, even in individuals without any psychiatric diagnosis, do genetic risks for psychiatric disorders associate with the language-math performance gap? To test this, we studied the associations of polygenic scores for the six psychiatric disorders with language and math performances only in the controls using multiple linear regression analysis. Both math and language grades were included in the same regression model to measure the correlation of language grades with polygenic risk for psychiatric disorders after controlling for math grades and vice versa.

We found that the polygenic score associations were in the same direction as the corresponding phenotypic associations. The results suggested that individuals who were not diagnosed with psychiatric disorders, but with a higher polygenic risk for psychiatric disorders, performed better in language relative to math (Fig. 4b; Supplementary Table 12). With regard to SCZ and BD, we observed an inverse association pattern between phenotypic analyses and polygenic score analyses (Fig. 4a,b). The SCZ cases had significantly poorer math grades, but not better language grades compared to controls (math: Beta = − 0.44; SE = 0.03; P = 3 × 10^–33^; language: Beta = 0.01; SE = 0.03; P = 0.64). However, individuals without SCZ, but with a higher polygenic risk for SCZ had significantly poorer math grades as well as better language grades (math: Beta = − 0.07; SE = 0.01; P = 1.4 × 10^–11^; language: Beta = 0.10; SE = 0.01; P = 5.1 × 10^–16^). This pattern was inverse for BD. The BD cases had significantly poorer math grades as well as better language grades compared to controls (math: Beta = − 0.37; SE = 0.04; P = 1 × 10^–16^; language: Beta = 0.24; SE = 0.04; P = 8.5 × 10^–8^). However, individuals without BD, but with a higher polygenic risk for BD had better language grades, but not poorer math grades (math: Beta = − 0.002; SE = 0.01; P = 0.88; language: Beta = 0.12; SE = 0.02; P = 2 × 10^–8^). A similar inverse pattern between SCZ and BD has been known with regard to genetic correlations with educational attainment and intelligence. SCZ shows a significant negative genetic correlation with intelligence, but a small positive genetic correlation with educational attainment^18^ (also E1) whereas BD shows a significant positive genetic correlation with educational attainment^10^ (also E1) but no genetic correlation with intelligence. The results point to a unique inverse relationship of SCZ and BD with cognition, though the molecular mechanisms underlying this relationship are unclear.

### Replication in TEDS

To replicate our findings, we analyzed 4547 genotyped individuals from the Twins Early Development Study (TEDS) cohort^33^ for whom General Certificate of Secondary Education (GCSE) school grades in English, math and science were available. The profile of the school grades in TEDS was similar to the one in iPSYCH: school grades were from the end of compulsory schooling; individuals were aged 15–16 years at the time of the examinations; the school grades were available in both math and language exams.

**Figure 5:**
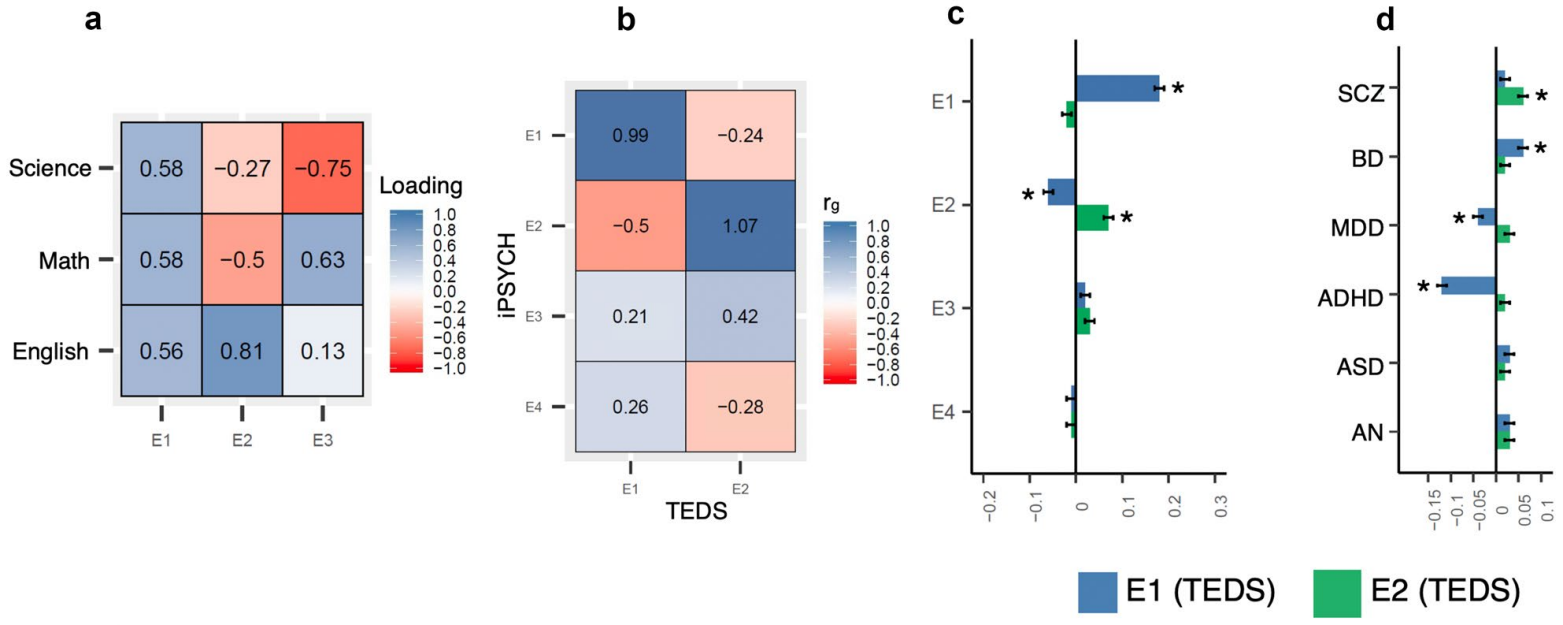
Replication analysis in TEDS. (**a**) Pearson correlations of E-factors with subject-specific school grades; (**b**) Genetic correlations of TEDS E-factors with iPSYCH E-factors estimated using bivariate LD score regression; (**c**) Associations of polygenic scores for E1, E2, E3 and E4 (based on iPSYCH SNP weights) with E1 and E2 in TEDS tested using linear regression; standardized effect sizes (beta) and standard errors are shown; (**d**) Associations of polygenic scores for the six psychiatric disorders with E1 and E2 in TED; standardized effect sizes (beta) and standard errors are shown. *SCZ* schizophrenia, *BD* bipolar disorder, *MDD* major depressive disorder, *ADHD* attention deficit hyperactivity disorder, *ASD* autism spectrum disorder, *AN* anorexia nervosa. Star symbols indicate statistical significance after multiple testing corrections (P < 0.006 for panel c and P < 0.004 for **d**).

PCA of English, math and science grades in TEDS yielded subject loadings that were similar to the subject loadings in the iPSYCH: E1 (first PC) had similar positive loadings from all three subjects; E2 (second PC) had positive loading from English and negative loadings from math and science; importantly, the English (0.81) and math (− 0.50) loadings were higher than science loading (− 0.27) suggesting that E2 captured mainly math and language performances (Fig. 5a). The factors E1 and E2 explained 83.4% and 10.4% of the variance in the school grades in TEDS respectively. Since the grades were not broken down to written and oral exams or a grade in a foreign language that was taken by everybody was not available (unlike Denmark, where the English exam is compulsory and so taken by everybody), we could not derive factors in TEDS equivalent to E3 and E4 in the iPSYCH.

We performed GWASs of E1 and E2 in TEDS and tested their genetic correlations with E1 and E2 in iPSYCH. The factors E1 and E2 in the two cohorts correlated almost completely (E1: r_g_ = 0.99; SE = 0.13; P = 3.3e−13; E2: r_g_ = 1.07; SE = 0.77; P = 0.16; Fig. 5b; Supplementary Table 13). The genetic correlation of E2 between the cohorts, however, was not statistically significant due to the relatively smaller sample size of the TEDS. Nevertheless, when we predicted E1 and E2 in TEDS using polygenic scores (constructed using the effect sizes from GWASs of E1 and E2 in iPSYCH), we observed significant associations supporting the genetic correlation analyses (Fig. 5c; Supplementary Table 14).

We also tested the genetic associations of E1 and E2 with the psychiatric disorders in TEDS using polygenic score analysis (Fig. 5d; Supplementary Table 15). The polygenic scores for the six psychiatric disorders were associated with E1 and E2 in TEDS in the same directions as the corresponding associations in iPSYCH. Notably, we observed positive associations between E2 and all six psychiatric disorders, albeit most of the associations except SCZ were only borderline significant; SCZ showed the strongest association with E2 (Beta = 0.06; SE = 0.01; P = 3.6 × 10^–06^). Hence, overall, we observed an agreement in the results between iPSYCH and TEDS that the genetic variants associated with E2 (better performance in language relative to math) were also associated with increased risk for psychiatric disorders, especially SCZ.

### Association of E2 with creativity

**Figure 6:**
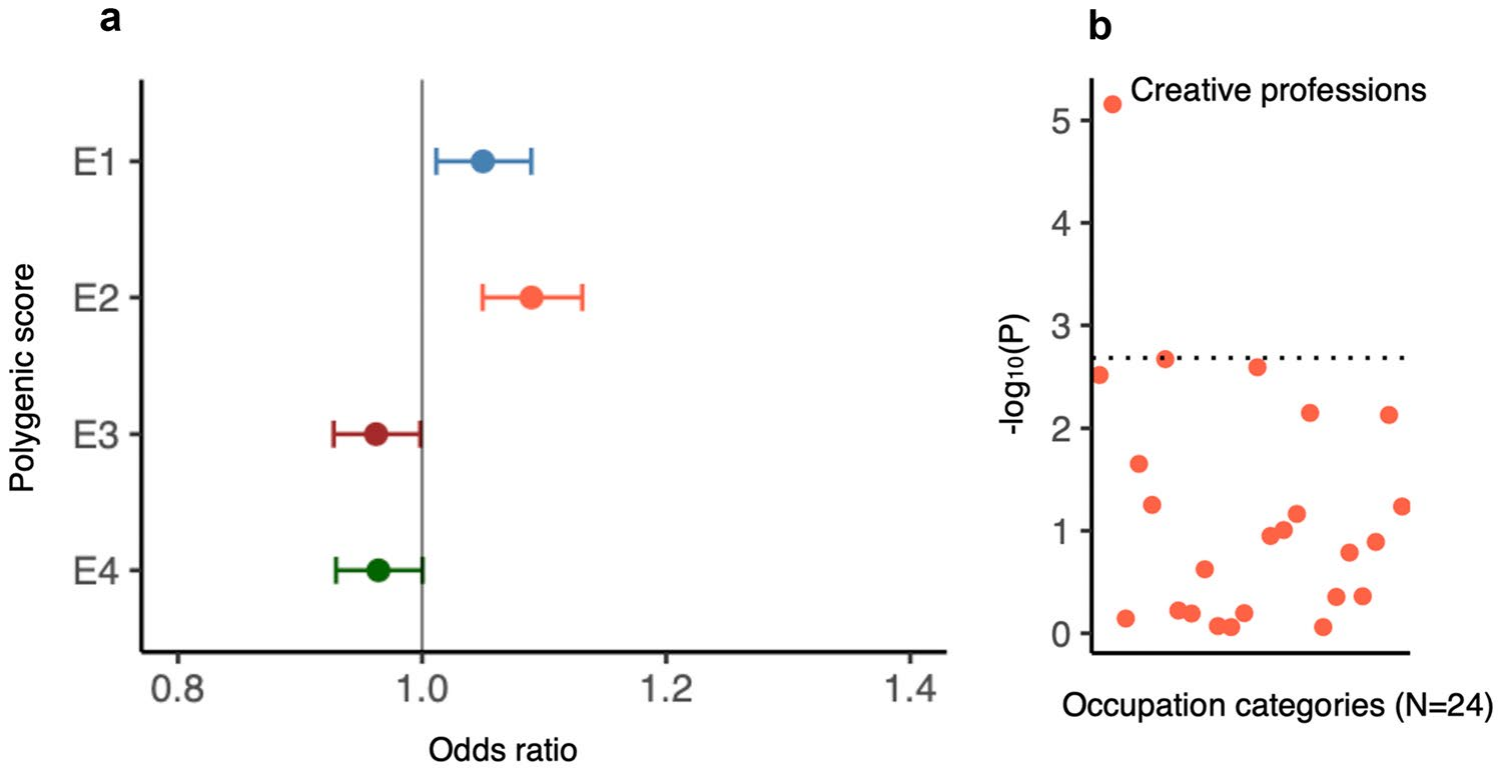
Genetic associations of E-factors with creativity. (**a**) Associations of polygenic scores for E-factors with creative professions (‘arts, design, entertainment, sports and media’ vs rest) in MVP tested using logistic regression; odds ratio and 95% confidence intervals are shown; (**b**) Associations of polygenic scores for E-factors with all 24 occupation categories in the MVP tested using logistic regression; Negative log10 P values are shown; the dotted line statistical significance threshold after multiple testing correction (P = 0.002).

Creativity has been historically believed to relate positively with psychopathology^34^. Many epidemiological studies^35–37^ and genetic studies^38,39^ have reported supporting findings. Our analyses showed that E2 was associated with increased risks for multiple psychiatric disorders as well as with increased language performance. Hence, we asked if common variants associated with E2 also associate with creativity. To evaluate this we analyzed 167,575 individuals from the Million Veterans Program (MVP) biobank^40^, for whom information on occupation category was available. We classified individuals employed in the category ‘arts, design, entertainment, sports and media’ as creative professionals and the rest as controls. We constructed polygenic scores for all four E-factors using effect sizes from the iPSYCH GWASs and compared the scores between the creative professionals and controls after adjusting for their highest level of education, age and sex along with other covariates (“Methods”). For sensitivity analysis, we also compared the scores of individuals in each of the other occupation categories against the rest. We found that the E2 polygenic scores were significantly higher in creative professionals compared to others. This association was specifically observed between E2 and creative occupation. That is, among the four E-factors, E2 showed the strongest association with creativity, and among 24 occupation categories, creative occupations showed the strongest association with E2. (Fig. 6; Supplementary Table 16). The results suggested that individuals with a higher genetic predisposition to perform better in language relative to math in school (indexed by a higher polygenic score for E2) have higher odds of being employed in a creative occupation later in life.

## Discussion

To our knowledge, the results presented here represent the most comprehensive report to date on the phenotypic and genetic differences in subject-specific school performances in individuals with and without psychiatric disorders. The study has been possible due to the unique register-based resources linked to the iPSYCH^25^ and ANGI^26^ cohorts, which enabled us to combine genetic information of the study individuals with their school grades in the Danish education ﻿register^27^. Our cohort is the largest available to date for the study of the genetics of school grades. Our study poses notable advantages compared to previous GWASs of educational attainment^13,28^. The phenotype, school grades, is fine-grained and hence captures more variance, for example, multiple individuals with the same number of years of education could differ substantially in their school grades. Our phenotype is objectively measured (graded by the teachers) unlike educational attainment, which is mostly self-reported and hence likely to have more imprecision and heterogeneity compared to school grades. Our study participants were all from a single large cohort hence the study variables—school grades and psychiatric diagnoses—are likely to have less heterogeneity compared to meta-analytic studies where different cohorts follow different methods of ascertainment. Our study individuals were identified through nationwide registers and hence not subject to voluntary participation bias that has been shown to affect GWAS results, particularly the genetic correlations^41^.

The availability of grades in multiple subjects for the same individuals enabled us to perform a PCA and decompose the school grades into distinct factors (E1, E2, E3, and E4) each representing unique cognitive abilities such as math and language. We replicated the PCs E1 and E2 in an independent cohort, TEDS^33^, and demonstrated near-perfect genetic correlations for E1 and E2 between iPSYCH and TEDS, thereby showing that the latent factors E1 and E2 are robust and reproducible.

Factor E1 measured the overall school performance and showed a strong genetic correlation with educational attainment^28^. Importantly, both E1 and educational attainment showed similar genetic correlations with the six psychiatric disorders. The results suggest that genetic relationships of psychiatric disorders with cognitive function inferred based on phenotypes (educational attainment and fluid intelligence) measured later in life (i.e., timepoints typically beyond the age of onset of most psychiatric disorders) are highly similar to the relationships inferred based on phenotypes (school performance) measured in early in life (i.e., timepoints before the age of onset most psychiatric disorders, except the ones with childhood-onset—ASD and ADHD). Although the results do not inform about causality or its direction, they inform that the observed genetic relationships are not merely a reflection of the effects of psychiatric disorders on cognitive and socioeconomic outcomes later in life.

The main findings of our study are those related to E2 as they offer novel insights into the relationship between psychiatric disorders and cognition. E2 measured the language performance relative to math performance and showed significant positive phenotypic associations with all six psychiatric disorders, which were supported by genetic correlations and/or polygenic score associations. Further analysis of actual language and math grades confirmed that the positive associations of E2 with the six psychiatric disorders are indeed due to increased language and decreased math performances in the cases compared to controls. Replication analysis in TEDS further validated the findings. In the TEDS cohort, the polygenic scores for all six psychiatric disorders showed associations with E2 in the positive direction. However, the association was statistically significant only for SCZ, which is likely due to the relatively smaller sample size in TEDS.

The E2 findings add two important insights. Firstly, the findings suggest that the positive genetic correlations of ASD^8^, BD^10^ and AN^12^ with educational attainment reported by earlier GWAS were likely driven by language-specific cognition. Though individuals with higher genetic risk for ASD or BD or AN are genetically predisposed to attain higher education as suggested by the positive genetic correlation with educational attainment, we speculate that they do so by choosing a field that requires language skills rather than math skills. GWASs of educational attainment separately in language and math-related fields in the future might help to confirm our hypothesis.

Secondly, the positive genetic association between E2 and risk for psychiatric disorders suggests that the genetic variants associated with increased risk for psychiatric disorders are associated with poor math ability or better language ability or possibly both. It is however unclear if the positive association between psychiatric risk and language ability is a true biological link. If so, this association might have an evolutionary basis as has been suggested previously, particularly in the context of SCZ^42,43^. Alternatively, the association of psychiatric risk variants with increased language ability could be simply due to that the individuals with the risk variants compensate for their math deficits by being good in non-math subjects including language.

Our final analysis suggested that individuals genetically predisposed to perform relatively better in language than in math at school more often choose a creative occupation later in life such as writing, acting and music. As we have demonstrated that these individuals were also at risk for psychiatric disorders, the finding aligns with two previous studies that demonstrated that individuals with increased genetic risk for SCZ and BD are more often involved in creative occupations^38^ or score better in creativity tests compared to the general population^39^. However, it is unclear if the association with creative occupations indicates that the individuals with the risk variants become creative due to their better language skills or simply choose creative professions as such professions suit well to their relatively poor math skills.

Our study has the following limitations. First, our interpretations assume that school performances in language and mathematics exams capture the cognitive abilities related to language and math domains respectively. However, our assumptions may not be entirely true as other factors including family^44^ and school socioeconomic statuses^45^ influence students’ school performances. Furthermore, like any other GWASs^13,14^, the effect sizes that we report are likely to be inflated as we haven’t accounted for the rearing environment^44^. Future studies that use a within-family design^46^ might help address these limitations. Second, although our primary sample, iPSYCH, has a better sampling design and do not suffer from participation bias^41^, the individuals included in the current study are only a subset and are not representative of the full iPSYCH cohort. We included only those who were functional enough to go to school and attend the exams. Hence, our study sample is slightly biased towards better-functioning individuals and as a result, many of our findings cannot be generalized to the disorders, for example, findings related to ASD might apply only to the high-functioning subtypes. Third, not all the individuals in the case groups received their diagnoses before the exams. This factor was not accounted for in our analyses since the diagnoses were register-based and it is therefore not possible to confirm if the individuals were asymptomatic before the date when the diagnoses were first registered. Fourth, as our sample is highly enriched for psychiatric disorders, we included psychiatric diagnoses as covariates in the GWASs. Recent studies have shown that including heritable covariates in GWAS might lead to collider bias^47^. However, this is less likely in our case as we have replicated the genetic correlation findings in only controls using polygenic score analysis and in addition, we also replicated the findings in an independent cohort, which is representative of the general population.

In summary, through an extensive analysis of subject-specific school grades in a large sample, we have convincingly demonstrated for the first time that individuals with psychiatric disorders exhibit wide differences in their math and language cognitions. These differences seem to have a genetic basis understanding which is important as the knowledge may help to treat the cognitive deficits associated with these disorders.

## Online methods

### Study cohorts

Our study individuals come from four cohorts: iPSYCH^25^, ANGI^26^, TEDS^33^ and MVP^40^. The main analyses were performed in iPSYCH and ANGI cohorts. iPSYCH is a large Danish case cohort established for the study of genetic and environmental risk factors associated with major psychiatric disorders (ADHD, ASD, MDD, SCZ, BD). ANGI is an international collaboration established between scientists in the United States, Australia/New Zealand, Sweden and Denmark to establish a cohort of individuals with and without AN. The ANGI participants involved in our study were those recruited in Denmark along with the iPSYCH participants. Hence, they were processed along with iPSYCH samples. All further descriptions about sample recruitment, genotyping and ethical approvals of the iPSYCH cohort apply to ANGI as well. The phase-1 release^25^ of the iPSYCH cohort, after QC, comprises 77,639 individuals—51,101 with one or more of the six disorders and 27,605 controls—who were identified from a baseline sample comprising of the entire Danish population (N = 1,472,762) born between 1981 and 2005. The cases were selected based on the psychiatric diagnosis information in the Danish Psychiatric Central Research Register^48^ and Danish National Hospital Register^49^. The controls were randomly sampled from the baseline sample. In the current study, 30,982 genotyped individuals who had school grades information available in the Danish education register^27^ were included.

TEDS^33^ is a large twin cohort comprising more than 16,000 twin pairs who were born either in England or Wales between 1994 and 1996. The twins were around 18 months old at the time of recruitment and were followed up longitudinally. Information on cognitive abilities, educational attainment, behaviour and emotion was collected. Among the TEDS participants, 4547 individuals, comprising one of each of the twin pairs, who were genotyped and also had information on their GCSE school grades were included in the current study.

The MVP^40^ is a large biobank at the Department of Veterans Affairs (VA), USA, established to study genetic and environmental influences on human diseases and traits. The participants being recruited into the study are active users of the US Veterans healthcare system. The MVP v3.0 data release comprised 455,789 genotyped individuals. Information on demographics, health, lifestyle, medical history and family history were collected through questionnaires or through electronic health records in the US Veterans healthcare system. Among those in the MVP v3.0 release, 165,575 individuals, who were identified to be of European ancestries and provided information on occupation and educational attainment, were included in the current study.

### Ascertainment of psychiatric disorders in the iPSYCH cohort

The psychiatric diagnoses of the iPSYCH case samples were identified through Danish Psychiatric Central Research Register^48^ and Danish National Hospital Register^49^. The diagnosis codes are based on the International Classification of Diseases, 10th revision. The ICD-10 codes of the six disorders as follows: ADHD—F90.0; ASD—F84.0, F84.1, F84.5, F84.8 or F84.9; MDD—F32-39 [Since 96% of the individuals had either F32 (depressive episode) or F33 (recurrent depressive disorder), we call it as MDD rather than as affective disorders]; BD—F30-31; SCZ—F20; AN—F50.0. Individuals with mental retardation (ICD10 F70-79) were excluded.

### School grades in the iPSYCH cohort

The school grades in the iPSYCH cohort were from the exit exam (also called as ninth-level exam or FP9) given at the end of compulsory schooling in Denmark. The exam grades were obtained from Danish education registers^27^ that maintain school grades from all public schools in Denmark since 2001.

We chose three subjects namely Danish, English and math for the current study. These subjects were chosen because they were compulsory, and so were available for a maximum number of individuals in the cohort. The examination types included written, oral and grammar in Danish, oral in English, and either written and oral or problem-solving with and without aids in math. The data were from the exams conducted between 2002 and 2016.

The grades were on a seven-point scale: − 3 (unacceptable performance), 00 (inadequate performance), 02 (adequate performance), 4 (fair performance), 7 (good performance), 10 (very good performance) and 12 (excellent performance). The seven-point grading system was followed only since 2007. Before 2007, a ten-point grading system was followed. The grades included in our study, from the years 2002–2006, were converted to seven-point grades using the conversion table provided by the Danish Ministry of Education (https://ufm.dk/en/education/the-danish-education-system/grading-system/old-grading-scale). A minimum score of 02 is considered as passing grade. The grade 00 is given if the student has handed over a blank paper or performed extremely poorly. Absentees without reason were graded − 3. Absentees with an acceptable reason, for example, acute illness, are not graded but are given an opportunity to take the exam in the subsequent year.

We applied strict sample-level quality control based on the school grade data. We removed individuals who were either younger than 14.5 years or older than 17.5 years at the time of the examinations. The common age group of the students taking the ninth-level exams in Denmark is 15 and 16 years. When multiple grades were available for a student, we considered only the grade obtained on the first attempt. We included only individuals who had grades in all the examinations in Danish, English and math chosen for the current study. Also, we removed individuals who had grades in different subjects from different years to avoid heterogeneity that may arise if the student was taught by different teachers, was at different schools or had different peers between the years.

### School grades in the TEDS cohort

The school grades in the TEDS cohort were from the GCSE exams given at the end of compulsory schooling in UK^33,50^. The grades were available in three compulsory subjects namely English, math and science. Unlike iPSYCH data, under each subject only one grade was available. The GCSE grades were self-reported by either the participants or their parents. A validation of the self-reported school grades in TEDS has been performed previously by correlating with the grades extracted from the national pupil database (NPD) (https://data.gov.uk/dataset/9e0a13ef-64c4-4541-a97a-f87cc4032210/national-pupil-database) for a subset of the individuals, which showed that the self-reported grades correlate highly (> 0.95) with NPD grades^33^. The GCSE grades ranged between four (G; lowest grade) to 11 (A+; highest grade), with four being the lowest pass grade.

### Occupation and educational attainment in the MVP cohort

Information on primary occupation was collected from all MVP participants through a questionnaire. Only the category of occupation was collected. The participants chose one of the 24 categories (Supplementary Table 17) that matched the best with their primary occupation at the time of recruitment. Individuals who chose the category: ‘Arts, Design, Entertainment, Sport and Media’ were considered creative professionals, and the rest as controls. The highest level of education achieved by the participants was also collected through a questionnaire. The participants chose one of the following answers: less than high school; high school diploma/GED; some college credit, but no degree; associate’s degree (e.g., AA, AS); bachelor’s degree (e.g., BA, BS); master’s degree (e.g., MA, MS, MBA); professional or doctoral degree. The categories are then converted to the number of years of education following International Standard Classification of Education (ISCED) 1997 guidelines (Supplementary Table 18).

### Genotyping and Imputation in the iPSYCH cohort

The source of DNA for genotyping was dried blood spot—two punches of diameter 3.2 mm equivalent to a volume of 6 µl of blood^25^. The blood spots of the iPSYCH participants were taken from the Danish neonatal screening biobank, which stores dried blood spots, taken 4–7 days after birth from the heel of the neonate, for all individuals born in Denmark since 1981^51,52^. The blood spots were matched with the individuals using the unique identification number that is used across all the registers in Denmark. The extracted DNA was then whole genome amplified in triplicates before genotyping. The genotyping was performed using Illumina Infinium PsychChip v1.0 array. Following standard QC of the genotyped markers (e.g., call rate > 0.98, MAF > 0.01, Hardy–Weinberg equilibrium P value > 1 × 10^–6^), phasing and imputation was carried out. Phasing was performed using SHAPEIT3^53^ and imputation was performed using IMPUTE2^54^ with 1000 genomes phase-3 as the reference panel.

### Genotyping and imputation in the TEDS cohort

The Source of DNA for genotyping in the TEDS cohort was saliva collected at the time of recruitment^33^. The DNA extracted from the saliva was genotyped using either Illumina HumanOmniExpressExome chip or Affymetrix Gene Chip 6.0. Following standard QC procedures, the genotyped markers are phased using EAGLE-2^55^, followed by imputation using MaCH^56^ with Haplotype reference consortium (release 1.1)^57^ as the reference panel. Both phasing and imputation were performed through the Sanger imputation services^58^. Imputation was performed separately for individuals genotyped using Illumina and those genotyped using Affymetrix. The genotyping chip used was accounted for in the genetic analysis by including a dummy variable for the two chips as a covariate.

### Genotyping and imputation in the MVP cohort

The Source of DNA for genotyping in the MVP cohort was peripheral venous blood collected at the time of recruitment or during the follow-up visits^40,59^. A genotyping chip, called MVP chip (modified Affymetrix Axiom Biobank array), was specifically designed for the MVP biobank. The MVP chip contains 723,000 markers enriched for exome SNPs, validated tag SNPs for diseases including psychiatric disorders, and variants specific to African American and Hispanic populations. Phasing and imputation were performed using either MaCH^56^ or minimac^60^ and SHAPEIT3^53^ or IMPUTE2^54^ respectively.

### Relatedness and population stratification in the iPSYCH cohort

All the individuals involved in the current study were unrelated and had European ancestries. Related pairs of individuals were identified using identity by descent (IBD) analysis using Plink v1.90^61^. One of each related pair (PIHAT > 0.20) was randomly excluded. PCA was performed in the unrelated individuals using approximately 23,000 imputed variants of high quality (imputation info score > 0.90, MAF > 0.05, missing rate < 0.01 and LD independent [r^2^ < 0.1]). Among the study individuals, a subset whose parents and paternal and maternal grandparents were born in Denmark were identified based on the Danish civil register. These individuals were used as a reference group to identify population outliers. The first five PCs of the reference individuals were used to construct a five-dimensional ellipsoid with a diameter of eight standard deviations (calculated from the PCs). Those individuals who fell outside the ellipsoid were considered non-Europeans and excluded from the study.

### Relatedness and population stratification in the TEDS cohort

Within each of the genotyped twins, one individual was randomly selected for the current study. Relatedness was estimated among those selected using IBD analysis using Plinkv1.90^61^ and one of each related pair (PIHAT > 0.125) was further removed randomly. PCA analysis was performed using EIGENSTRAT^62^ for the unrelated individuals after merging with the 1000 genomes EUR samples. With the 1000 genomes EUR samples as reference, ancestry outliers were removed iteratively based on the first 10 PCs.

### Relatedness and population stratification in the MVP cohort

Relatedness between MVP participants was inferred using the kinship coefficient calculated using the software KING^63^. Related individuals are removed using a kinship coefficient cut-off ≥ 0.088. Individuals of European ancestries were identified using a machine learning algorithm called HARE^64^ that uses information about both self-reported ethnicities as well as principal components derived from PCA of genetic markers. The PCA was performed using EIGENSOFT v.6 (https://www.nature.com/articles/ng1847).

### PCA of school grades

PCA of school grades in the iPSYCH cohort was performed in R using the ‘principal’ function from the ‘psych’ R-package. The datasets from the years 1990–2006 (dataset 1) and 2007–2016 (dataset 2) were analyzed separately since the math grade types differed between the two. Both the PCAs yielded six PCs that explained 100% of the variance in the school grades. The PCs were rotated using simplimax algorithm^65^ and then used for the analysis. We focused on the first four PCs that explained ~ 90% of the variance in the school grades. The subject-specific loadings of the four PCs were similar between datasets 1 and 2. Also, the genetic correlations of the four PCs in dataset 1 with the corresponding PCs in dataset 2 were close to 1. Hence, we combined the PCs of both datasets and analyzed them together. The dataset origin of the PC values was coded as a binary variable and included as a covariate in all related analyses. PCA of school grades in the TEDS cohort was performed in R using ‘prcomp’ function from the base R-package.

### GWAS

The GWASs in the iPSYCH cohort were performed in Plink v.1.90^61^ using linear regression adjusted for age (age at the time of examination), sex, first 10 PCs, genotyping batches, group variable for PCA of school grades and psychiatric diagnoses. Totally 6,391,200 variants with MAF > 0.01 and INFO > 0.80 were included in the final analysis.

The GWASs in the TEDS cohort were performed in Plink v.1.90^61^ using linear regression adjusted for age, sex, first 10 PCs and genotyping chips. Totally 5,266,884 variants with MAF > 0.01 and INFO > 0.80 were included in the final analysis.

### Phenome-wide association analysis

The Phenome-wide association analysis was performed using GWAS atlas^66^, an online database of GWAS summary statistics. The GWAS atlas database holds summary statistics for 4571 GWASs (the number represents unique studies, but not unique phenotypes). Using variant identifier (RSID) of the index variants in the seven genome-wide loci, we queried the GWAS atlas and obtained all the associations with P value < 0.05. The phenotypes are provided along with category labels such as cognitive and psychiatric. All the associations under the cognitive category are provided in Supplementary Table 5 (educational attainment, though categorized as environmental, is also included in the list).

### SNP based heritability

The SNP-based heritability of the school grades and the E-factors was measured using genome-based restricted maximum likelihood (GREML) analysis implemented in the genome-wide complex trait analysis (GCTA) software^67^. A genetic relationship matrix (GRM) was constructed using around 7 million genetic variants (MAF > 0.01; INFO > 0.2; Missing rate < 0.95) for the whole iPSYCH cohort. The SNP-based heritability was then calculated for only the individuals included in this study (N = 30,982) using the GCTA-GREML analysis adjusted for the same covariates as the main GWAS.

### Polygenic scores derivation

In the iPSYCH cohort, polygenic scores for ASD^8^, ADHD^7^, SCZ^31^, BD^32^, MDD^11^, AN^26^, educational attainment^28^ and intelligence^14^ were derived using effect sizes from summary statistics of published studies with large sample sizes. The GWASs of ASD and ADHD, however, were based on predominantly the iPSYCH sample. Hence, the polygenic scores for ASD and ADHD were derived in-sample using a leave-one-out approach as described previously^7,8^. Briefly, we divided the full iPSYCH sample into ten groups and derived polygenic scores for ASD and ADHD in each group separately. The SNP weights required for polygenic score calculation in each group came from a GWAS of ASD and ADHD performed in the rest of the nine groups combined, thereby ensuring no sample overlap between training and target samples. The summary statistics were LD clumped using the 1000 genome EUR reference panel to identify LD-independent variants. The clumped summary statistics were then used for constructing polygenic scores using Plink v1.90^61^. Ten P-value thresholds (S1 = 5 × 10^−8^, S2 = 1 × 10^−6^, S3 = 1 × 10^−4^, S4 = 1 × 10^−3^, S5 = 0.01, S6 = 0.05, S7 = 0.1, S8 = 0.2, S9 = 0.5 and S10 = 1.0) were used, yielding ten polygenic scores for each trait. The polygenic score based on the threshold that gave the best prediction (i.e., explained the maximum variance) was used in the association analysis. We acknowledge that this method of generating polygenic scores, called ‘clumping and thresholding’ (C&T), has been superseded by newer methods that offer better prediction performances^68,69^. However, the C&T-based scores that we generated had enough predictive power in most cases to perform statistical association tests and infer the direction of the associations.

In the TEDS cohort, polygenic scores were constructed for six psychiatric disorders using the same summary statistics that were used for the polygenic score construction in the iPSYCH cohort. In addition, polygenic scores were constructed for E-factors using effect sizes from the GWASs of E-factors in iPSYCH. The summary statistics were LD clumped using 1000 genomes EUR reference panel to identify LD-independent variants. The clumped summary statistics were then used for constructing polygenic scores using PRSice v2.2.3^70^.

In the MVP cohort, polygenic scores were constructed for E-factors (E1, E2, E3 and E4) using effect sizes from the iPSYCH GWAS of E-factors. The summary statistics were processed using PRS-CS software^68^ to generate weights (posterior SNP effect sizes). Default settings were used for calculating weights using PRS-CS (γ-γ prior = 1; parameter b in γ-γ prior = 0.5; MCMC iterations = 1000; number of burn-in iterations = 500; thinning of the Markov chain factor = 5). Then, based on the derived weights individual-level polygenic scores for E-factors were calculated using Plink v2.0^71^ software.

### Polygenic scores analysis

The polygenic score associations were tested using either linear (if analyzing school grades) or logistic regression (if analyzing occupation category). The covariates used in the iPSYCH and TEDS cohorts were the same as the ones used in the corresponding GWASs. In the MVP cohort, the following covariates were used: age at recruitment, sex, first 20 ancestral PCs, genotyping batches and number of years of education. The variance explained by the polygenic scores was interpreted using *R*^*2*^ (if continuous outcomes: E-factors) or Nagelkerke pseudo *R*^2^ (if binary outcomes: occupation). Two regression models were constructed: one with the polygenic score and all covariates included (model 1) and the other with only the covariates included (model 2). The reported *R*^*2*^ values were calculated by subtracting the *R*^*2*^ of model 2 from *R*^*2*^ of model 1.

### Genetic correlations

The genetic correlations were calculated using either GCTA bivariate REML^67^ or LD score bivariate regression^30^. The genetic correlations between individual school grades, between the E-factors from dataset 1 and E-factors from dataset 2, and pairwise genetic correlations between the E-factors (after combing both datasets) were all calculated using GCTA using individual genotypes. The genetic correlations between the E-factors and other traits (years of education, intelligence and the six psychiatric disorders), and between the iPSYCH E-factors and TEDS E-factors were calculated using LD score regression using GWAS summary statistics. We used the precalculated LD scores available from the LD score regression website. Only the HapMap variants (SNP list available from the LD score regression website) were used for the analysis. The reference and effect alleles in the summary statistics are aligned with that of the HapMap list. Then, the summary statistics files were munged using the munge script from LDSC software (with default settings). The munged files were then used to estimate genetic correlations.

### Multiple testing corrections

The statistical significance of all the analyses was assessed after multiple testing corrections. The P value threshold in each of the analyses was decided based on the number of unique hypotheses tested. In the analysis of the heritability of E-factors, the P-value threshold was set to 0.01 (0.05/4). In the GWAS analysis, the P value threshold was set to 1.25 × 10^–8^ (5 × 10^–8^/4). In the analysis of genetic correlations and polygenic score associations of E-factors with educational attainment and intelligence, the P-value threshold was set to 0.006 (0.05/8). In the analysis of phenotypic associations, genetic correlations and polygenic score associations between the E-factors (N = 4) and the psychiatric disorders (N = 6), the P value threshold was set to 0.002 (0.05/24). In the analysis of phenotypic associations and polygenic score associations of math and language (N = 2) with psychiatric disorders (N = 6), the P-value threshold was set to 0.004 (0.05/12). In the analysis of genetic correlations and polygenic score associations of iPSYCH E-factors (N = 4) with TEDS E-factors (N = 2), the P value threshold was set to 0.006 (0.05/8). In the analysis of polygenic score associations of TEDS E-factors (N = 2) with psychiatric disorders (N = 6), the P value threshold was set to 0.004 (0.05/12). In the analysis of polygenic score associations of E2 (N = 1) with occupations (N = 24), the P value threshold was set to 0.002 (0.05/24).

### Ethics approval and consent to participate

All the analyses in the iPSYCH data are within the permissions received from the Danish Scientific Ethics Committee, the Danish Health Data Authority, the Danish data protection agency and the Danish Neonatal Screening Biobank Steering Committee^25^. The iPSYCH project is based on individuals recruited via Danish registers, and obtaining informed consent from the participants has been exempted by the Danish ethical committee in accordance with the Act of Research Ethics Review of Health Research Projects (in Danish: Komitéloven), Section 10(1) (https://ipsych.dk/en/data-security/health-research-and-ethical-approval/). The analyses in the TEDS cohort are within the permissions received from the King’s College London Ethics Committee (reference: PNM/09/10-104)^33^. Parental consent was obtained for all the TEDS participants before data collection. All the participants in the MVP cohort have given written informed consent at the time of recruitment. The analysis involving MVP data in the current study is approved by the VA Central Institutional Review Board. We confirm that all the analyses we report in this manuscript were performed in accordance with relevant guidelines/regulations as recommended by the respective ethics committees.

## Supplementary Information

Supplementary Information 1.

Supplementary Tables.

Supplementary Information 3.

Supplementary Information 4.

## Data Availability

The GWAS summary statistics are available for download at https://ipsych.dk/en/research/downloads/.
