# Supplementary material for: Genome-wide association study of school grades identifies genetic overlap between language ability, psychopathology and creativity
Source: Sci Rep. 2023 Jan 9;13:429. doi: 10.1038/s41598-022-26845-0 (PMC9829693; doi:10.1038/s41598-022-26845-0)
Supplement: Supplementary file 3 — Supplementary Information 3. [file 41598_2022_26845_MOESM3_ESM.pdf]

Supplementary dataset 1 l Regional association plots

The regional association plots of the seven genome-wide loci (four for E1, two for E2 and one for E3) are in this file. The plots are made using online tool, LocusZoom (locuszoom.org). Genotypes of 1000g Phase 3 European subset were used for LD calculation.

In all the plots, in the X axis basepair positions (hg19), in the left Y axis $-\log_{10}$ P values and in the right Y axis recombination rates are plotted. The index variant (i.e. the most significant variant) is shown as a diamond and is labelled. The points are colored according to their LD (r^2) with the index variant. The genes located within the regions are displayed as gene tracks (thick line correspond to exon and thin line, intron). The arrowed end correspond to the 3' end of the gene.

GWAS of E1, Locus 2q11.2

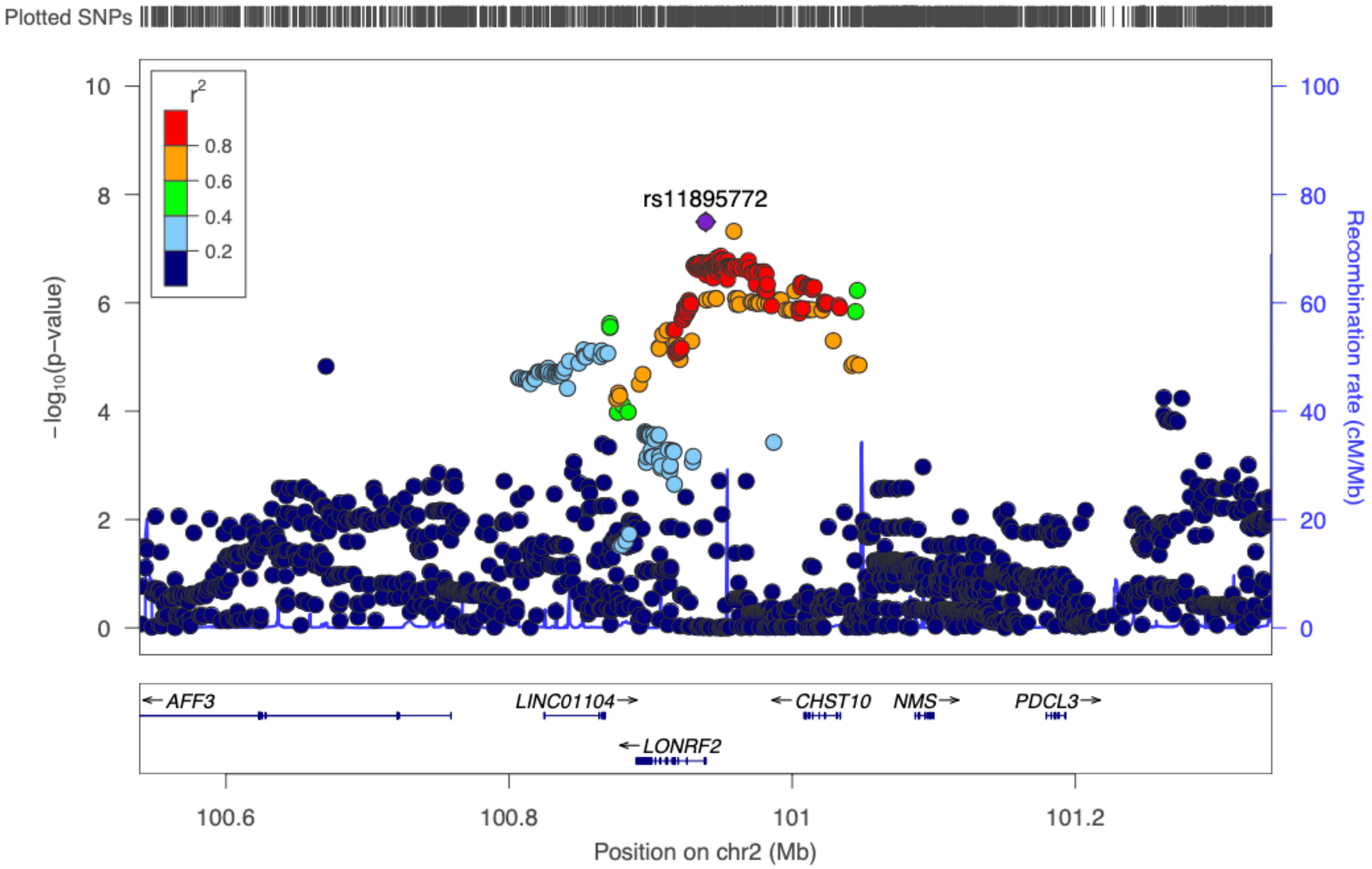

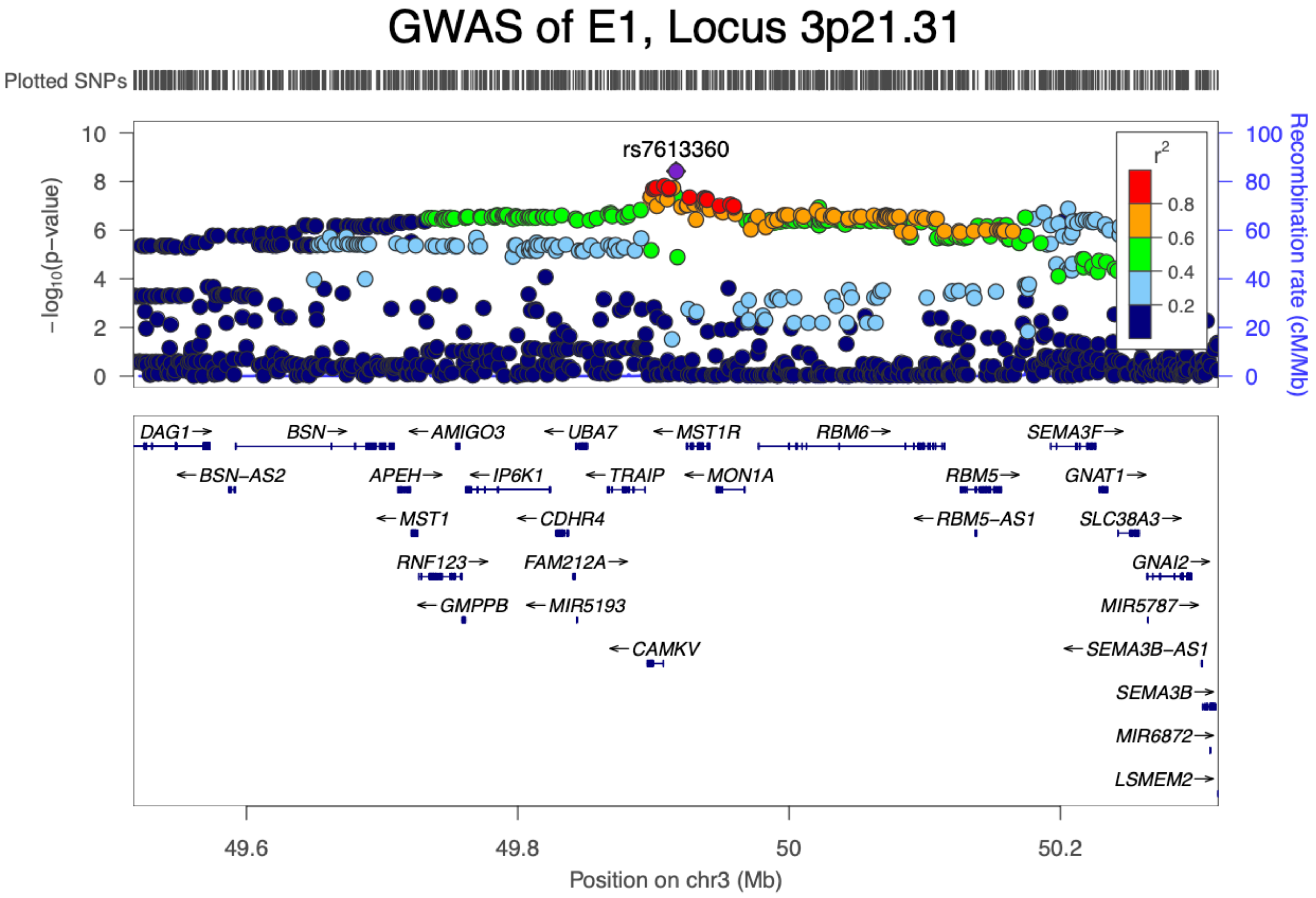

GWAS of E1, Locus 3p21.31
Plotted SNPs
rs7613360
r²
0.8
0.6
0.4
0.2
−log₁₀(p−value)
Recombination rate (cM/Mb)
10
8
6
4
2
0
100
80
60
40
20
0
DAG1→
BSN→
←AMIGO3
←UBA7
←MST1R
RBM6→
SEMA3F→
←BSN-AS2
APEH→
←IP6K1
←TRAIP
←MON1A
RBM5→
GNAT1→
←MST1
←CDHR4
←RBM5-AS1
SLC38A3→
RNF123→
FAM212A→
GNAI2→
←GMPPB
←MIR5193
MIR5787→
←CAMKV
←SEMA3B-AS1
SEMA3B→
MIR6872→
LSMEM2→
49.6
49.8
50
50.2
Position on chr3 (Mb)

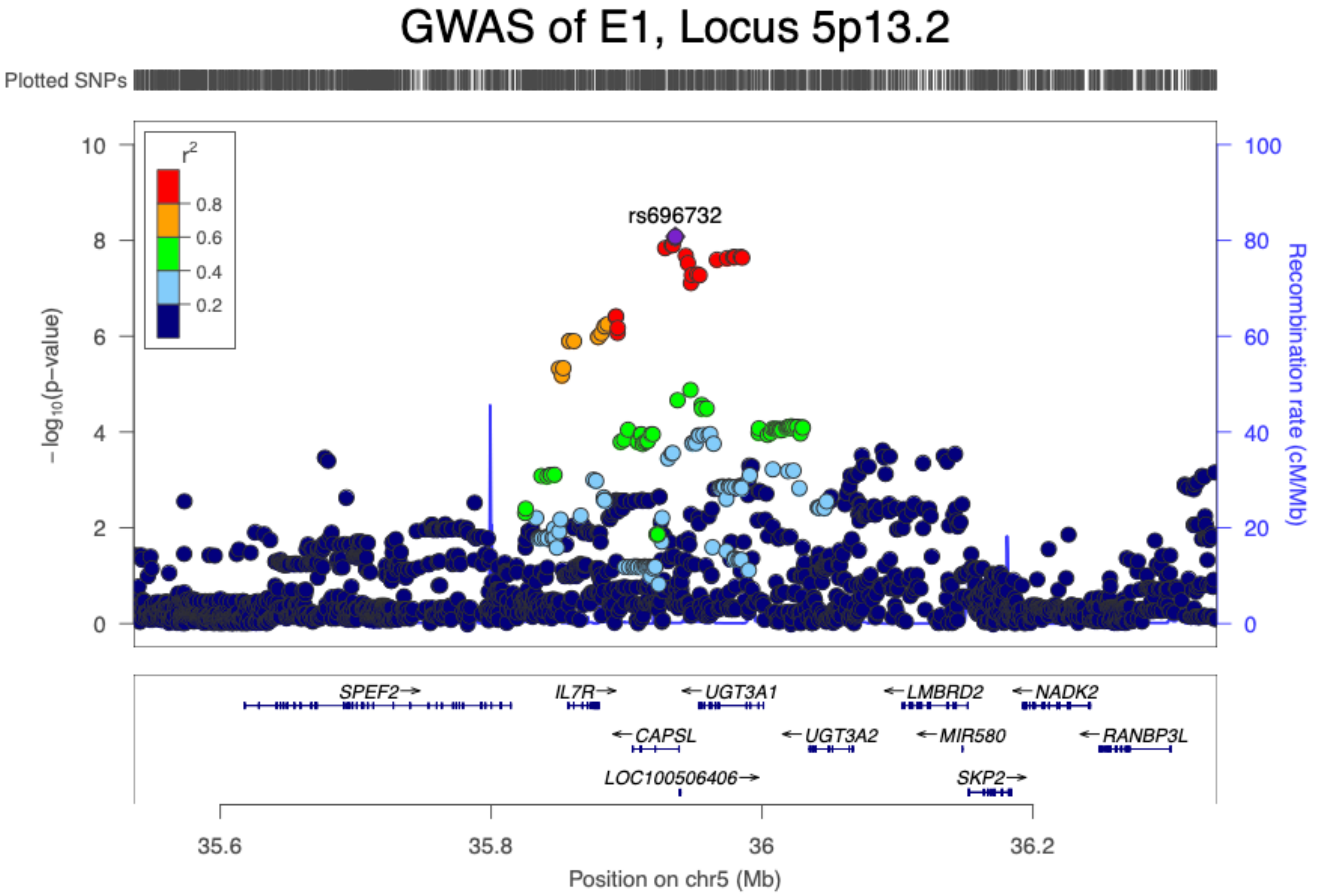
GWAS of E1, Locus 5p13.2
Plotted SNPs
rs696732
r2
0.8
0.6
0.4
0.2
10
8
6
4
2
0
-log10(p-value)
100
80
60
40
20
0
Recombination rate (cM/Mb)
SPEF2
IL7R
UGT3A1
LMBRD2
NADK2
CAPSL
UGT3A2
MIR580
RANBP3L
LOC100506406
SKP2
35.6
35.8
36
36.2
Position on chr5 (Mb)

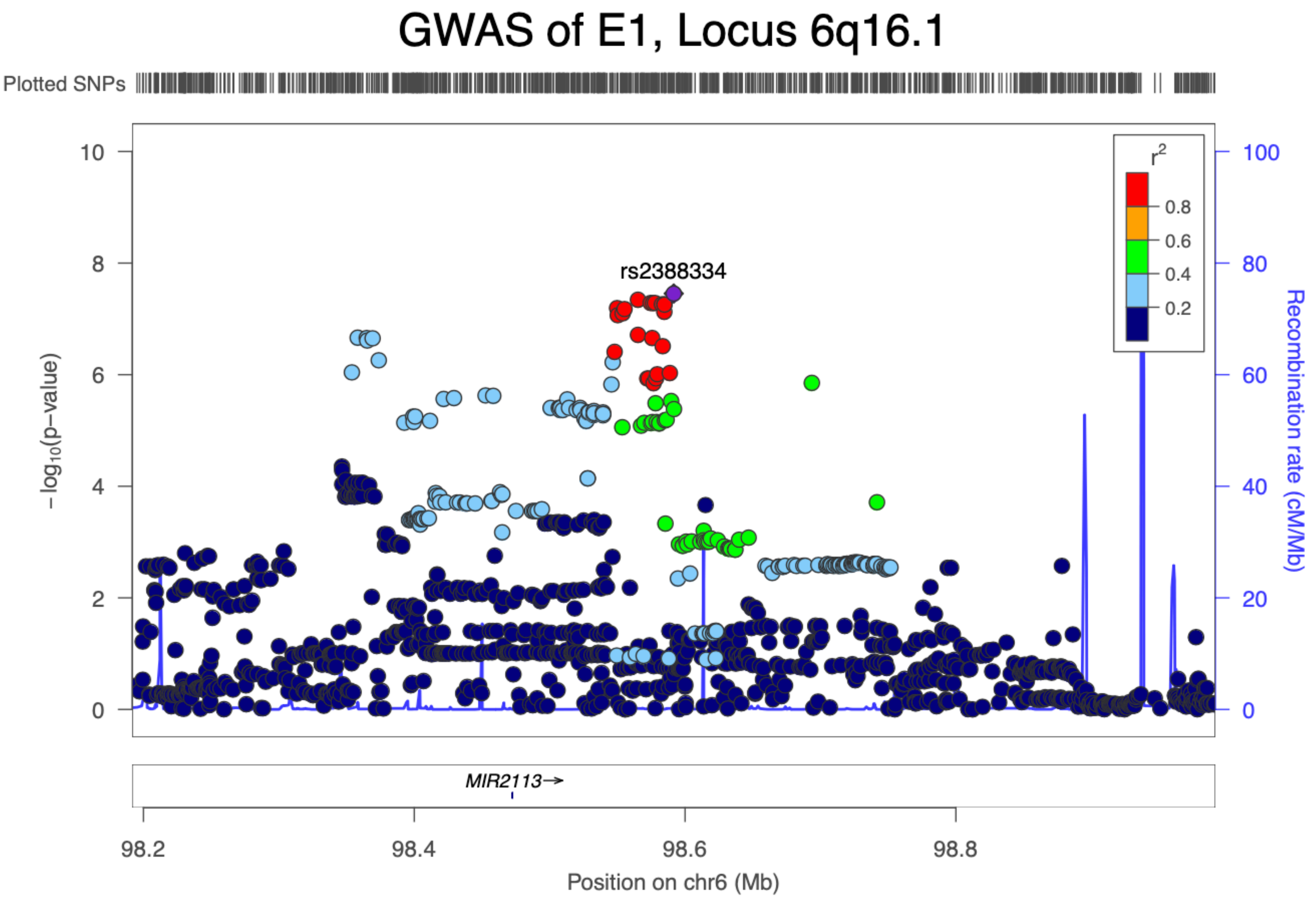
GWAS of E1, Locus 6q16.1
Plotted SNPs
rs2388334
r2
0.8
0.6
0.4
0.2
−log10(p−value)
Recombination rate (cM/Mb)
10
8
6
4
2
0
100
80
60
40
20
0
MIR2113→
98.2
98.4
98.6
98.8
Position on chr6 (Mb)

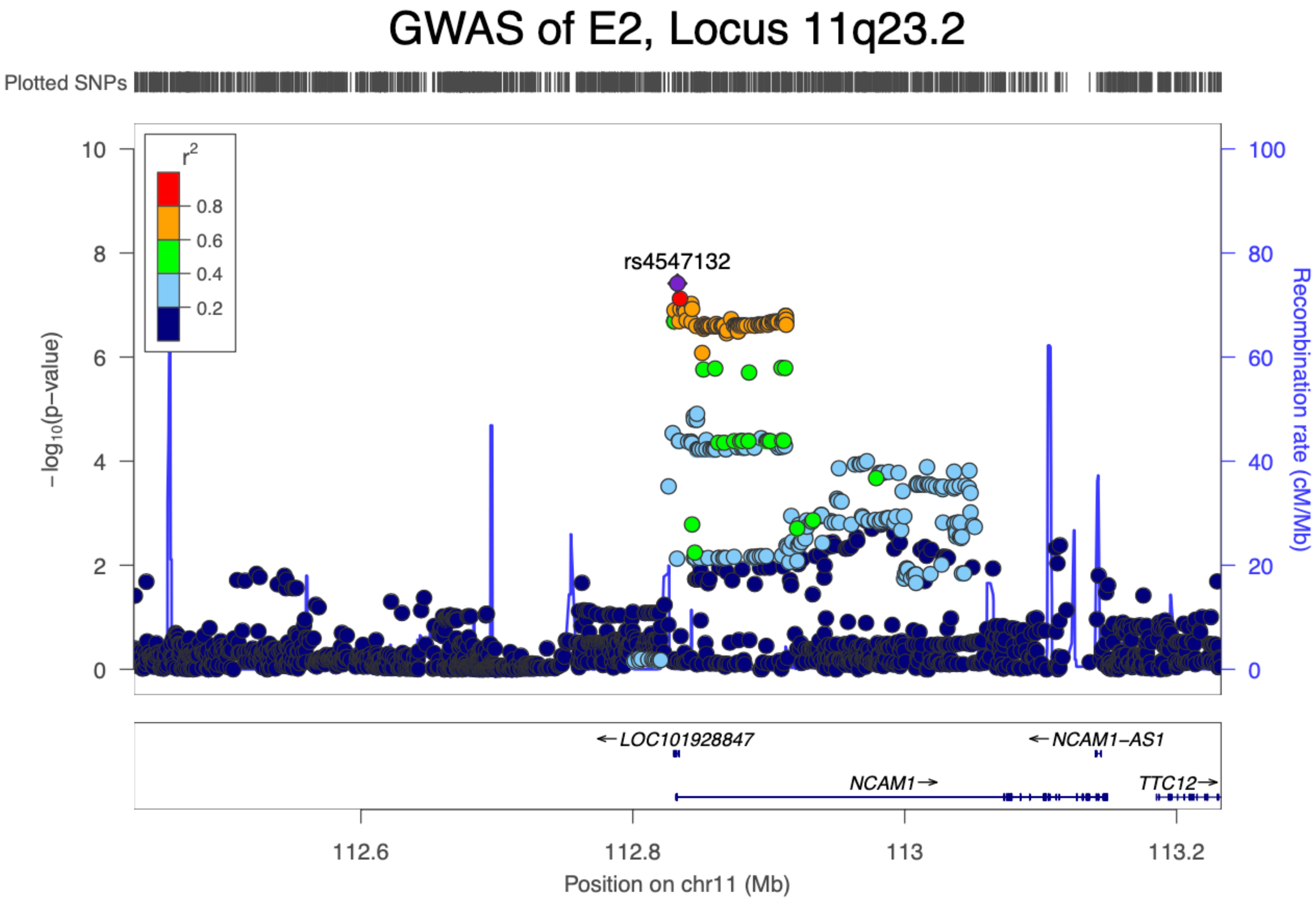

GWAS of E2, Locus 11q23.2
Plotted SNPs
rs4547132
r2
0.8
0.6
0.4
0.2
−log10(p−value)
Recombination rate (cM/Mb)
10
8
6
4
2
0
100
80
60
40
20
0
←LOC101928847
←NCAM1−AS1
NCAM1→
TTC12→
112.6
112.8
113
113.2
Position on chr11 (Mb)

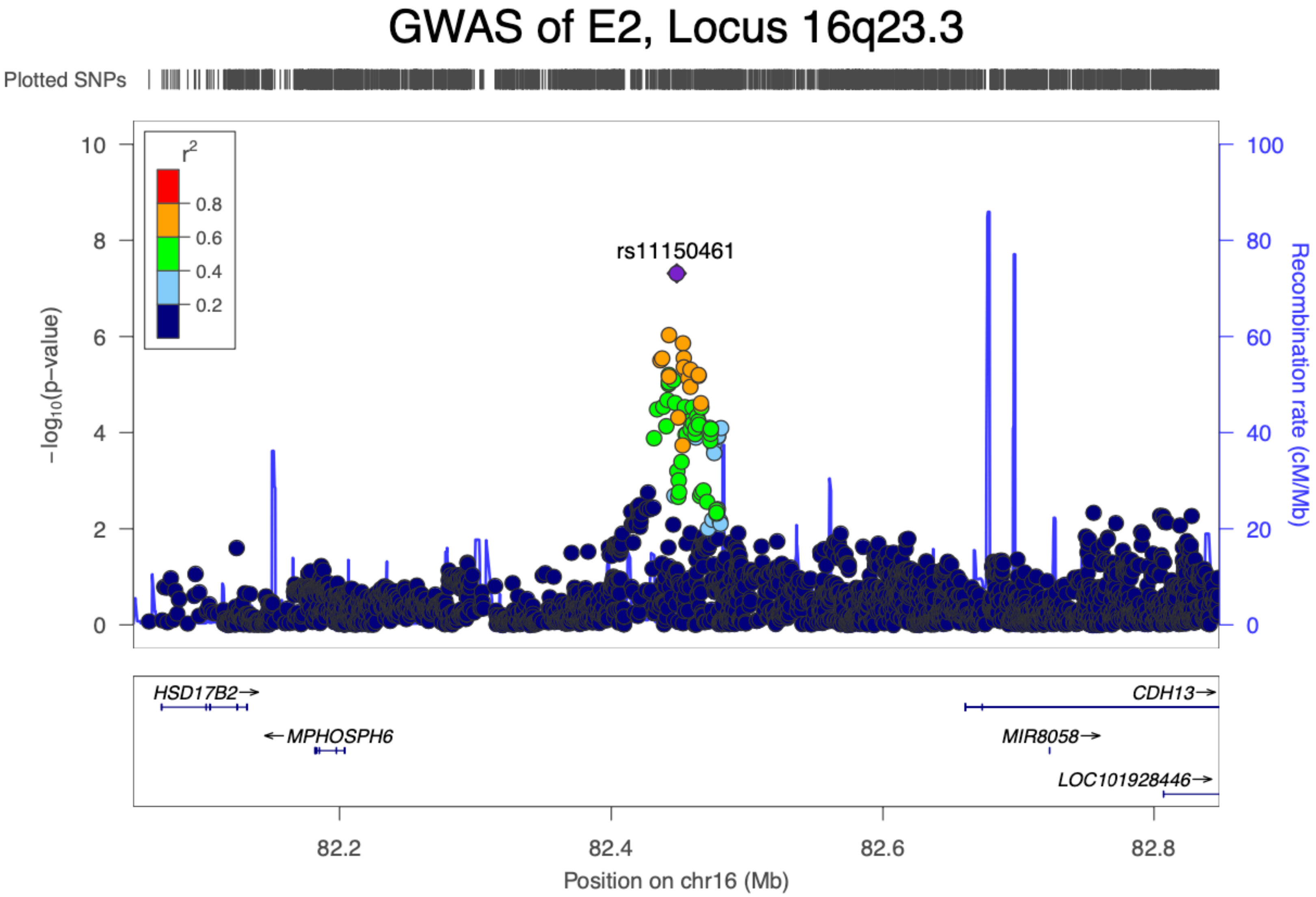

GWAS of E2, Locus 16q23.3
Plotted SNPs
r²
0.8
0.6
0.4
0.2
rs11150461
−log₁₀(p−value)
Recombination rate (cM/Mb)
HSD17B2
MPHOSPH6
CDH13
MIR8058
LOC101928446
82.2
82.4
82.6
82.8
Position on chr16 (Mb)

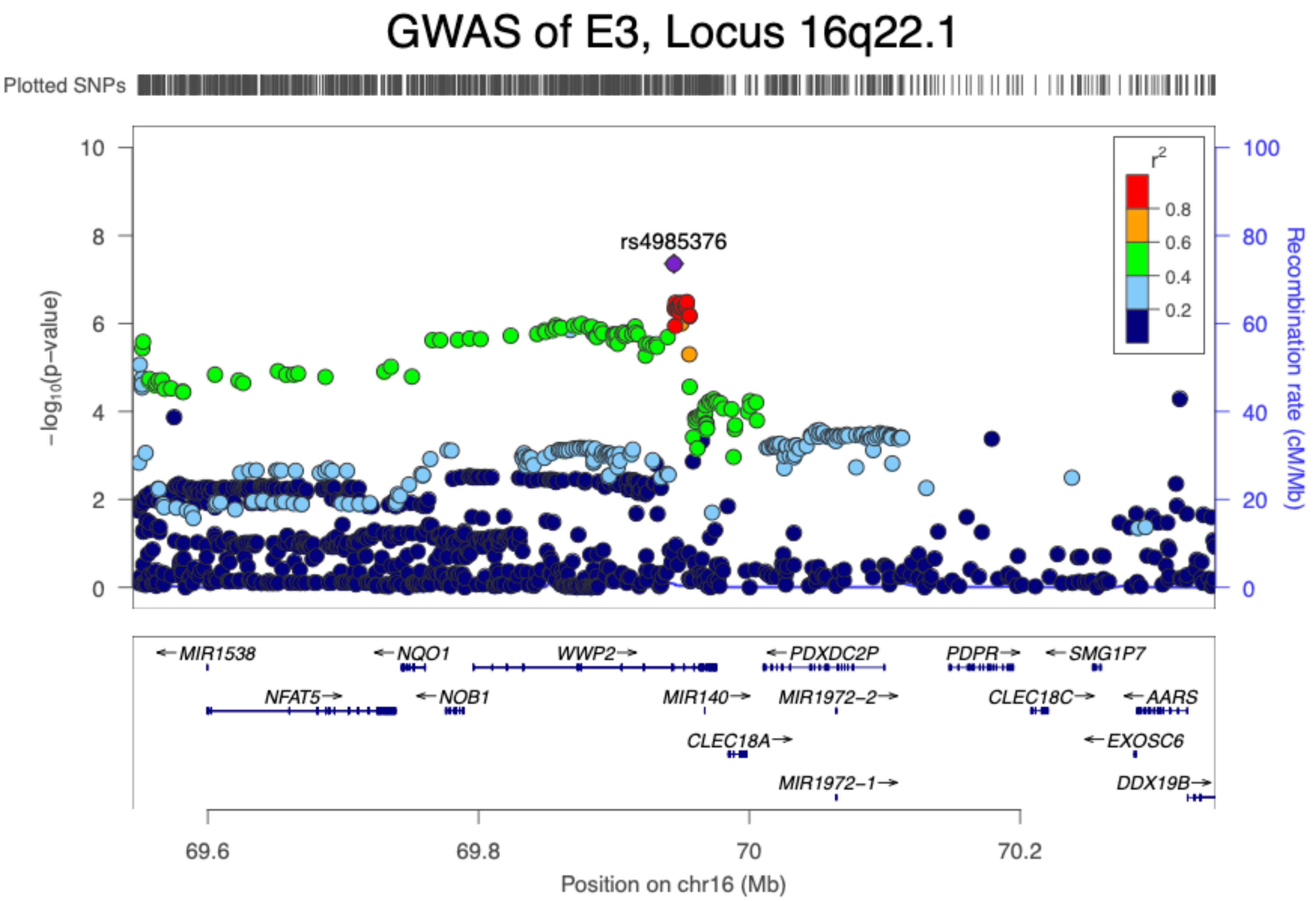
GWAS of E3, Locus 16q22.1
Plotted SNPs
rs4985376
-log10(p-value)
Recombination rate (cM/Mb)
r2
0.8
0.6
0.4
0.2
10
8
6
4
2
0
100
80
60
40
20
0
MIR1538
NQO1
WWP2
PDXDC2P
PDPR
SMG1P7
NFAT5
NOB1
MIR140
MIR1972-2
CLEC18C
AARS
CLEC18A
EXOSC6
MIR1972-1
DDX19B
69.6
69.8
70
70.2
Position on chr16 (Mb)
