# Supplementary material for: Genome-wide association study of school grades identifies genetic overlap between language ability, psychopathology and creativity
Source: Sci Rep. 2023 Jan 9;13:429. doi: 10.1038/s41598-022-26845-0 (PMC9829693; doi:10.1038/s41598-022-26845-0)

Supplementary dataset 2 | Phenome-wide association plots.

The PheWAS plots of seven variants are in this file. The seven variants are index variants in the genome-wide loci identified in this study--four for E1, two for E2 and one for E3. The PheWAS was done using GWASatlas online tool (atlas.ctglab.nl). Totally 4756 phenotypes were included. Only associations that are statistically significant after multiple testing correction ($P<1.05x10^{-5}$) were shown in the plots. Not all points can be labelled. Hence, only relevant phenotypes or phenotypes considered as interesting were labelled.

Phenotype: E1
Variant ID: rs7613360

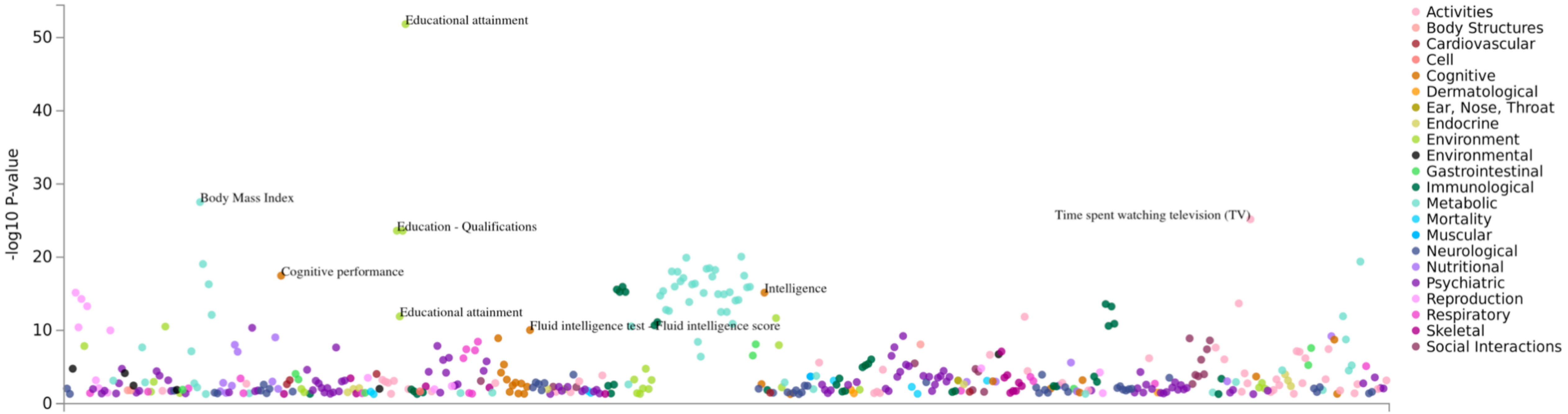

Phenotype: E1
Variant ID: rs2388334

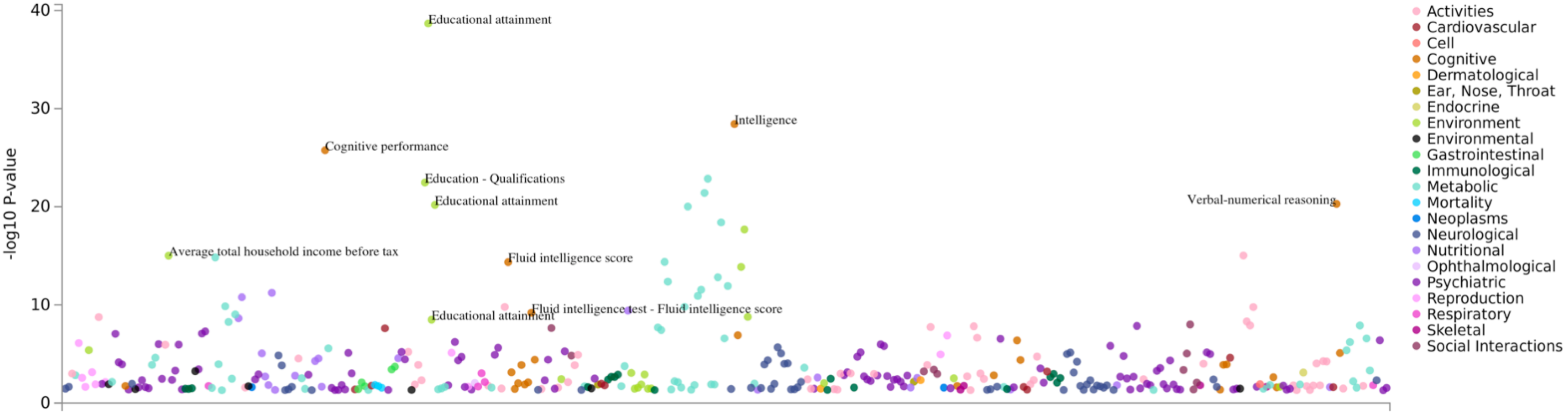

Phenotype: E1
Variant ID: rs11895772

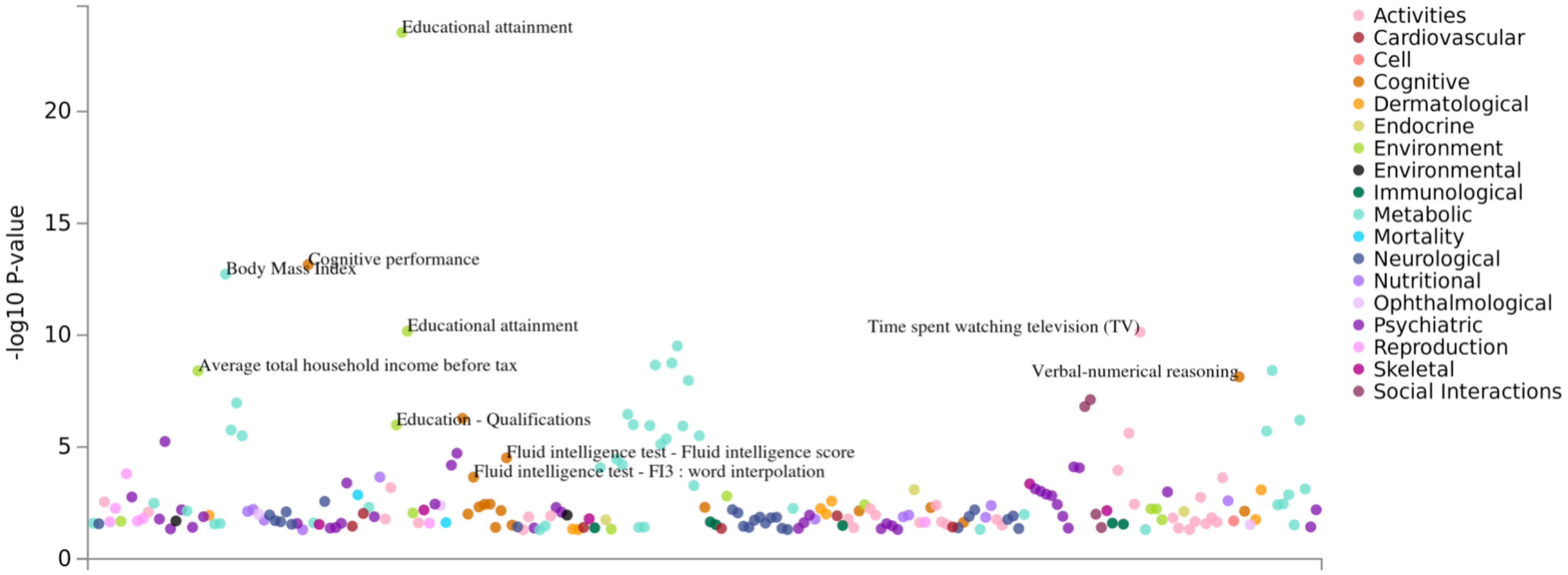

Phenotype: E1
Variant ID: rs696732

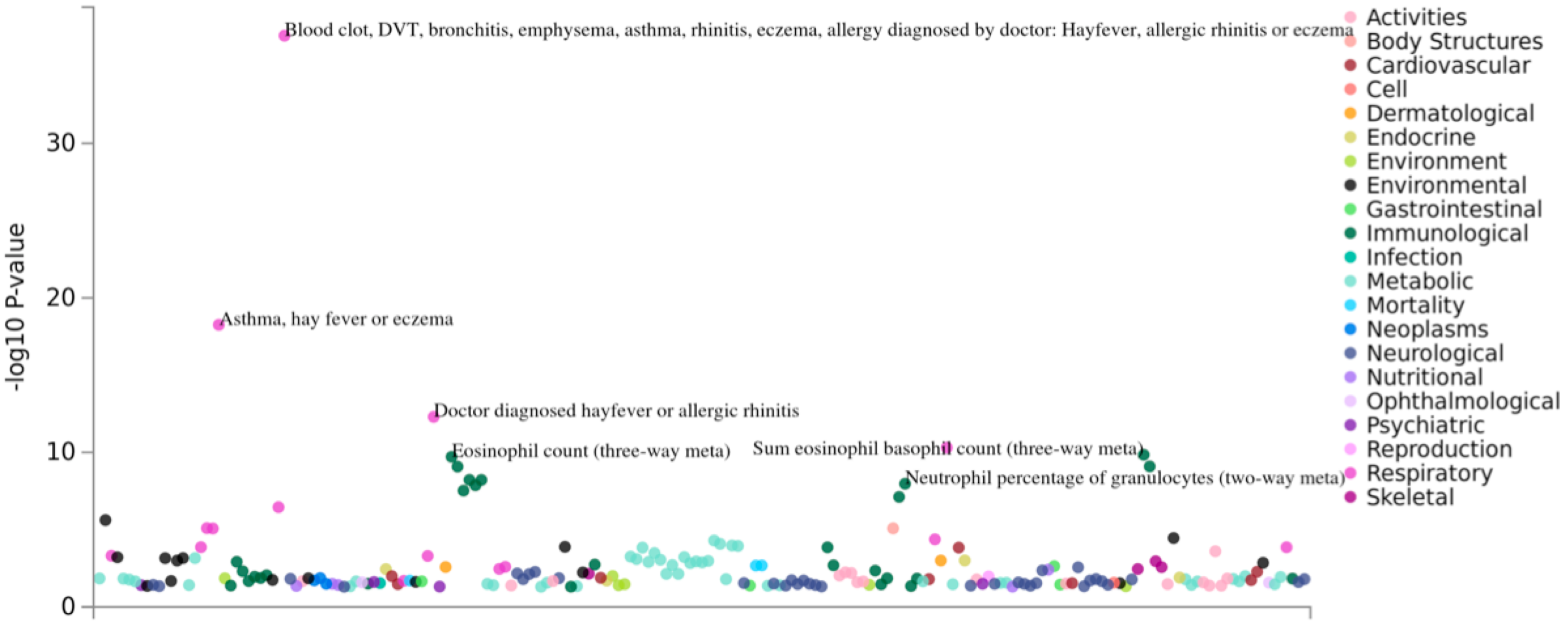

Phenotype: E2
Variant ID: rs4547132

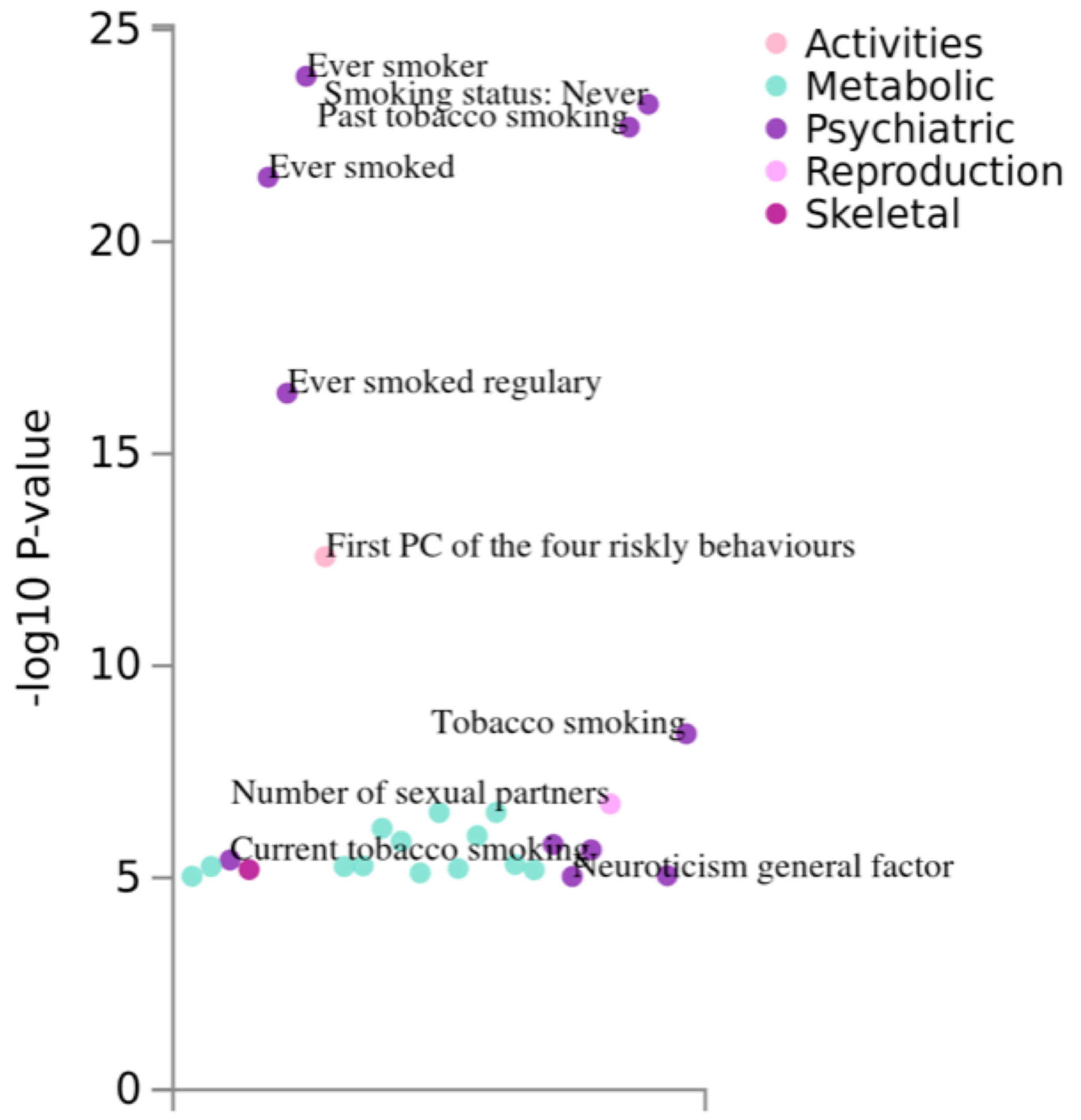

Phenotype: E2
Variant ID: rs11150461

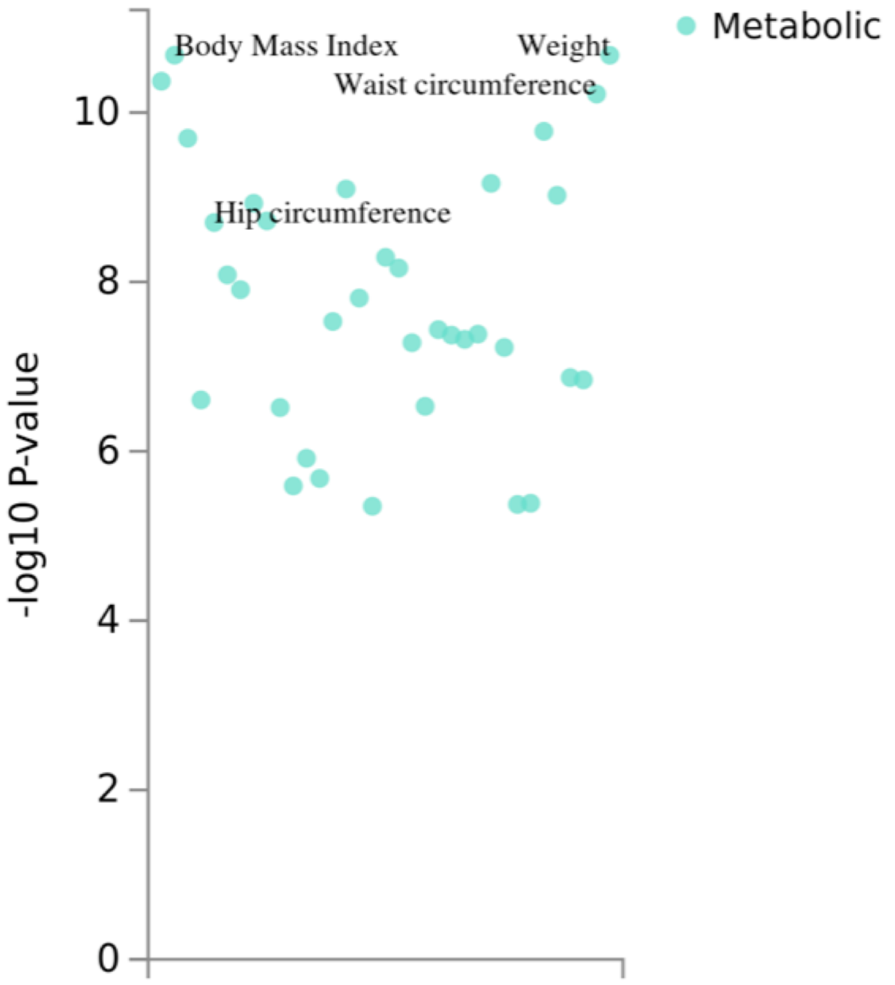

Phenotype: E3
Variant ID: rs696732

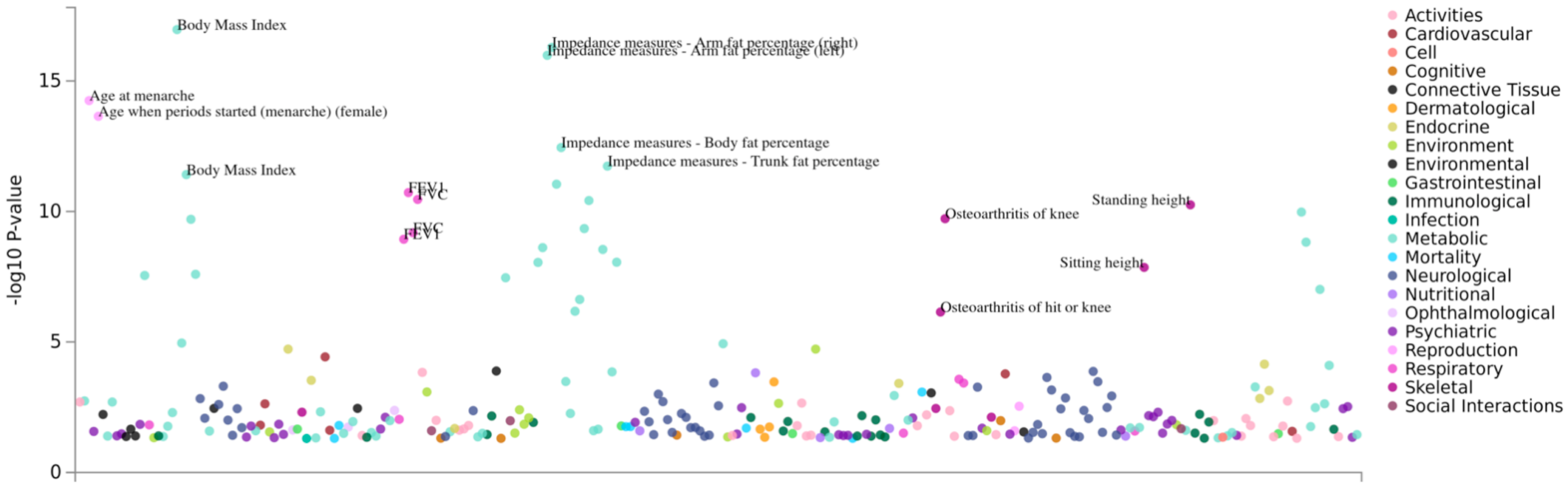

Supplement: Supplementary file 4 — Supplementary Information 4. [file 41598_2022_26845_MOESM4_ESM.pdf]
